# Transcriptomic analysis of *Stropharia rugosoannulata* reveals carbohydrate metabolism and cold resistance mechanisms under low-temperature stress

**DOI:** 10.1186/s13568-022-01400-2

**Published:** 2022-05-14

**Authors:** Haibo Hao, Jinjing Zhang, Shengdong Wu, Jing Bai, Xinyi Zhuo, Jiaxin Zhang, Benke Kuai, Hui Chen

**Affiliations:** 1 State Key Laboratory of Genetic Engineering and Fudan Center for Genetic Diversity and Designing Agriculture, Institute of Plant Biology, School of Life Sciences, Fudan University, 413 Room, No. 2005, Songhu Road, YangPu District, Shanghai, 200438 China; 2 National Research Center for Edible Fungi Biotechnology and Engineering, Key Laboratory of Applied Mycological Resources and Utilization, Ministry of Agriculture, Shanghai Key Laboratory of Agricultural Genetics and Breeding, Institute of Edible Fungi, Shanghai Academy of Agricultural Sciences, 309 Room, No. 1000, Jinqi Road, FengXian District, Shanghai, 201403 China

**Keywords:** *Stropharia rugosoannulata*, Transcriptomic, Carbohydrate enzymes, Antioxidant enzyme, Heat shock protein, Low-temperature stress

## Abstract

Low temperature is an important environmental factor that restricts the growth of *Stropharia rugosoannulata*; however, the molecular mechanisms underlying *S. rugosoannulata* responses to low-temperature stress are largely unknown. In this study, we performed a transcriptome analysis of a high-sensitivity strain (DQ-1) and low-sensitivity strain (DQ-3) under low-temperature stress. The liquid hyphae of *S. rugosoannulata* treated at 25 °C and 10 °C were analyzed by RNA-Seq, and a total of 9499 differentially expressed genes (DEGs) were identified. GO and KEGG enrichment analyses showed that these genes were enriched in “xenobiotic biodegradation and metabolism”, “carbohydrate metabolism”, “lipid metabolism” and “oxidoreductase activity”. Further research found that carbohydrate enzyme (AA, GH, CE, and GT) genes were downregulated more significantly in DQ-1 than DQ-3 and several cellulase activities were also reduced to a greater extent. Moreover, the *CAT1*, *CAT2*, *GR*, and *POD* genes and more heat shock protein genes (*HSP20*, *HSP78* and *sHSP*) were upregulated in the two strains after low-temperature stress, and the *GPX* gene and more heat shock protein genes were upregulated in DQ-3. In addition, the enzyme activity and qRT–PCR results showed trends similar to those of the RNA-Seq results. This result indicates that low-temperature stress reduces the expression of different AA, GH, CE, and GT enzyme genes and reduces the secretion of cellulase, thereby reducing the carbohydrate metabolism process and mycelial growth of *S. rugosoannulata*. Moreover, the expression levels of different types of antioxidant enzymes and heat shock proteins are also crucial for *S. rugosoannulata* to resist low-temperature stress. In short, this study will provide a basis for further research on important signaling pathways, gene functions and variety breeding of *S. rugosoannulata* related to low-temperature stress.

**Supplementary Information:**

The online version contains supplementary material available at 10.1186/s13568-022-01400-2.

## Introduction

*Stropharia rugosoannulata* Farl. ex Murrill, commonly known as the wine-cap *Stropharia* mushroom or king *Stropharia*, is a gorgeous mushroom with excellent taste and nutritional qualities, and it is widely distributed in northern temperature zones (Szudyga 1978; Yan and Jiang 2005). It is also a medicinal fungus with antitumor (Dai et al. 2009; Wang et al. 2018), antibacterial, antioxidant, antifatigue and blood glucose-lowering properties (Song et al. 2009; Yan and Xu 2018; Wu et al. 2019). *S. rugosoannulata* cultivation mainly uses various agricultural and forestry wastes, such as straw, tree branches and substrates of other edible fungi after cultivation (Yang et al. 2020). *S. rugosoannulata* was first domesticated in Germany in the 1960s, and it was recommended by the Food and Agriculture and Organization (FAO) as a cultivated mushroom for developing countries (Szudyga, 1978; de Oliveira et al., 2020, Yang et al. 2020). In the 1980s, *S. rugosoannulata* was introduced from Poland and successfully cultivated in China, although it was not promoted (Huang et al. 2010). Recently, due to the maturity of cultivation techniques and the development of recycling agriculture using microbial degradation straw, the cultivation of *S. rugosoannulata* has become very popular in China. However, large-scale cultivation can only be carried out in fields and woodlands, and it is cultivated in November and harvested from March to April of the second year (Ren et al. 2020). Therefore, the lower temperature during the overwintering process will cause stress to the hyphae, which will hinder the growth of the hyphae and affect the degradation of the substrate.

Low temperature is a very important environmental factor in the artificial cultivation of edible fungi (Sakamoto 2018). Low temperature threatens the life of fungal mycelia and is completely harmful in the vegetative growth stage of mycelia. However, low temperature is an important trigger for sexual reproduction in the reproductive growth stage of mycelia (Sakamoto 2018). Low-temperature stress stimulates the formation of fruit bodies and is widely used in the cultivation and production of many edible fungi, such as *Flammulina velutipes* (Sakamoto et al. 2002) and *Lentinus. edodes* (Nakazawa et al. 2008), *Pleurotus eryngii subsp. Tuoliensis* (Fu et al. 2016) and *Armillaria mella* (Ford et al. 2015). Notably, *S. rugosoannulata* is also subjected to low-temperature stress under rough cultivation conditions, and this adverse stress might hinder mycelial growth or stimulate fruit body formation. Thus, we need to investigate the mechanisms of the stress response of *S. rugosoannulata* under low-temperature stress.

The growth of organisms is easily limited by low temperatures due to the associated reduction in metabolic activity (Fürtauer et al. 2019). In plants, the reduction in amylase activity induced by low temperatures results in lower degradation of seed storage reserves, such as starch, thereby limiting the energy supply available for seed germination (Li et al. 2013). Low temperature can also reduce the growth rate and biomass of the fungus *Volvariella volvacea*, and 357 carbohydrate enzymes were found to be involved in the degradation of cellulose, hemicellulose and pectin under low temperature (Wang et al. 2009a; Bao et al. 2013). Laccase and peroxidase (POD) in the fungus *Pleurotus tuoliensis* appear to play a dominant role during nutrient growth under low-temperature induction conditions (Hua et al. 2018). Under low-temperature stress, fungi might also use different carbohydrate enzymes to convert the substrate into small molecular sugars to provide energy for the growth of mycelium. On the other hand, low temperatures can lead to the accumulation of reactive oxygen species (ROS) (Skyba et al. 2012), which may be another main reason for the decreased metabolic activity of mycelium. In general, the low-temperature tolerance of plants is considered connected to antioxidant defenses. For example, wheat resists low-temperature stress through superoxide dismutase (SOD), catalase (CAT) and ascorbate peroxidase (APX) (Li et al. 2013). In fungi, the antioxidant enzymes (SOD, CAT and APX) of *V. volvacea*, *Hypsizigus marmoreus* and *Penicillium sp.* are significantly increased under low-temperature stress (Wang et al. 2009a; Hu et al. 2009; Gocheva et al. 2006). In addition, heat shock proteins (HSPs) are reported to play an important role in protein folding when proteins are denatured due to oxidative stress, high pressure, and heat shock (Guo et al. 2018). Studies have also shown that low temperature can induce the expression of HSP genes in *Saccharomyces cerevisiae* and *V. volvacea* (Salvadó et al. 2012; Bao et al. 2013). Notably, the expression of the *Hsp90* gene in *P. tuoliensis* (Bailinggu) was significantly increased after low-temperature stress (Hua et al. 2018). This finding indicates that edible fungi will produce different functional proteins to resist low-temperature stress; however, the mechanism underlying the induction of the antioxidant response in *S. rugosoannulata* under low-temperature stress is poorly understood.

Transcriptome analysis is an important method of revealing gene expression and biological processes (Wang et al. 2009b), and it also represents an important method of studying the complex biological processes in edible fungi. For example, a transcriptome analysis of the parental strain and mutant strain of *V. volvacea* after low-temperature treatment revealed the mechanism of low-temperature tolerance (Lv et al. 2015), transcriptome analyses of *Pleurotus pulmonarius* after cold stress challenge revealed the cold response mechanism, and transcriptome and differentially expressed gene analyses at different developmental stages of *P. pulmonarius* treated with low temperature revealed the mechanism of low temperature-induced fruit body formation (Xie et al. 2018; Wang et al. 2020). Therefore, it is feasible to use transcriptome analysis to reveal the molecular biological mechanisms of *S. rugosoannulata*.

In this study, we used transcriptome technology to perform GO and KEGG enrichment analyses of *S. rugosoannulata* after low-temperature treatment. Furthermore, we analyzed the differential expression of genes that code for carbohydrate enzymes, antioxidant enzymes and heat shock proteins and determined the activities of related enzymes with roles at the physiological level. This study provides a theoretical basis for the study of the carbohydrate metabolism and cold resistance mechanisms in *S. rugosoannulata.*

## Materials and methods

### *S. rugosoannulata* mycelium cultivation and treatment

The *S. rugosoannulata strain* “DQ-1” (CGMCC5.2211) and “DQ-3” (CGMCC5.2223) were deposited in the China General Microbiological Culture Collection Center. The mycelia of the fungi were first incubated on potato dextrose agar plates at 25 ℃ for 4 days, and then the plates were cultured at 25 ℃ and 10 ℃ for 3 days and the growth rate was measured. In addition, the plates were punched out and inoculated in liquid medium containing 1% glucose, 0.5% rice straw powder, 0.3% peptone, 0.3% soybean meal, 0.1% MgSO_4_, and 0.15% KH_2_PO_4_ on a rotary shaker incubator at 150 rpm at 25 °C for 4 days, and then the mycelia were transferred to 25 °C and 10 °C Erlenmeyer flasks and treated for 3 days. The mycelia were harvested from the liquid cultures by sterile filtration, or samples were collected and dried at 65 ℃ to a stable biomass to determine the mycelial biomass, or then they were stored at – 80 ℃ for subsequent experiments. Three biological replicates of the mycelia were collected, and a total of 12 samples (SR1: DQ-1 mycelium treated at 25 ℃; SR3: DQ-3 mycelium treated at 25℃; SR6: DQ-1 mycelium treated at 10 ℃; and SR8: DQ-3 mycelium treated at 10℃) were used for the transcriptomic analysis.

### Total RNA isolation, cDNA library preparation, and Illumina sequencing

Total RNA was extracted from the hyphal samples using TRIzol® Reagent according the manufacturer’s instructions (Invitrogen, Carlsbad, CA, USA), and genomic DNA was removed using DNase I (Takara, Dalian, China). Then, RNA quality was determined using a 2100 Bioanalyzer (Agilent, Palo Alto, CA, USA) and quantified using an ND-2000 (NanoDrop Technologies) system (NanoDrop Technologies, Wilmington, DE, USA). High-quality RNA samples (OD260/280 = 1.8 ~ 2.2, OD260/230 ≥ 2.0, RIN ≥ 6.5, 28S:18S ≥ 1.0, > 10 μg) were used to construct a sequencing library. RNA-Seq transcriptome libraries were prepared using a TruSeqTM RNA Sample Preparation Kit from Illumina (San Diego, CA, USA) with 1 μg of total RNA. Briefly, messenger RNA was isolated with polyA selection by oligo(dT) beads and fragmented using fragmentation buffer. cDNA synthesis, end repair, A-base addition and ligation of the Illumina-indexed adaptors were performed according to Illumina’s protocol. Libraries were then size-selected to obtain cDNA target fragments of 200–300 bp on 2% Low Range Ultra Agarose, which was followed by PCR amplification using Phusion DNA polymerase (New England Biolabs, Ipswich, MA, USA) for 15 PCR cycles. After quantification by TBS380, paired-end libraries were sequenced by Illumina NovaSeq 6000 sequencing (150 bp*2, Shanghai BIOZERON Co., Ltd, Shanghai, China). All reads were deposited in the National Center for Biotechnology Information (NCBI) database under the BioProject accession number PRJNA758163.

### Read quality control and mapping

The raw paired-end reads were trimmed and quality-controlled by Trimmomatic (with parameters SLIDINGWINDOW: 4:15; MINLEN: 75) (version 0.36) (http://www.usadellab.org/cms/uploads/supplementary/Trimmomatic). Then, clean reads were separately aligned to the reference genome with orientation mode using hisat2 software (https://ccb.jhu.edu/software/hisat2/index.shtml). This software was used to map the default parameters. The quality assessment of these data was performed by qualimap_v2.2.1 (http://qualimap.bioinfo.cipf.es/), and htseq (https://htseq.Readthedocs.io/en/release_0.11.1) was used to count each gene read.

### Differential expression analysis and functional enrichment

To identify DEGs of the two *S. rugosoannulata* strains under the different temperature treatments, the expression level for each gene was calculated using the fragments per kilobase of exon per million mapped reads (FRKM) method. The R statistical package edgeR (Empirical analysis of Digital Gene Expression in R) (http://www.bioconductor.org/packages/release/bioc/html/edgeR.html/) was used for the differential expression analysis. The DEGs between two samples (SR1 vs. SR6, SR3 vs. SR8, SR1 vs. SR3 and SR6 vs. SR8) were selected using the following criteria: the logarithmic fold change was greater than 2 and the false discovery rate (FDR) was less than 0.05. To understand the functions of the differentially expressed genes, GO functional enrichment and KEGG pathway analyses were carried out by Goatools (https://github.com/tanghaibao/Goatools) and KOBAS (http://kobas.cbi.pku.edu.cn/home.do). DEGs were significantly enriched in GO terms and metabolic pathways when their Bonferroni-corrected *P value* was less than 0.05.

### Enzyme assay

The mycelial samples of *S. rugosoannulata* were ground into a fine powder in liquid nitrogen. Then, the samples (0.2 g) were homogenized in 1.8 mL of normal saline and centrifuged at 9000 ×*g* for 10 min. The supernatant was used to measure the cellulase, exo-1,4-beta glucanase, endo-1,4-beta glucanase, beta-glucosidase, superoxide dismutase (SOD) and catalase (CAT) activity with the corresponding assay kits (Comin Biotechnology, Suzhou, China).

Cellulase activity was measured according to the instructions of the assay kit. One unit of cellulase was defined as the amount of enzyme that decomposed sodium carboxymethyl cellulose to produce 1 μg of glucose, as monitored at 620 nm. In addition, the exo-1,4-beta glucanase, endo-1,4-beta glucanase, beta-glucosidase, SOD and CAT activities were measured according to a previously described method (Hao et al. 2019).

### Validation of gene expression by qRT–PCR

Total RNA was isolated from samples using TRIzol® reagent (Takara, Dalian, China) according to the manufacturer’s instructions. Approximately 2 μg of total RNA from *S. rugosoannulata* in the 12 samples (SR1, SR3, SR6 and SR8) was reverse-transcribed by M-MLV reverse transcriptase (Takara) using oligo (dT) as the primer. qRT–PCR was performed as described by Zhang et al. (2017) using SYBR (Takara, Dalian, China). The primers and internal reference gene (18S ribosomal RNA) are listed in Additional file 1: Table S1. In addition, the relative gene expression was analyzed using the 2^−ΔΔCt^ method described by Livak and Schmittgen (2001), and each experiment was performed in triplicate.

### Statistical analysis

All experimental data shown in this paper were based on three independent samples to ensure that the trends and relationships observed in the cultures were reproducible. The data and graphs were processed using GraphPad Prism 6.0. Differences among treatments were analyzed by one-way analysis of variance (ANOVA) combined with Duncan’s multiple range test at a probability of P < 0.05.

## Results

### Differences in the growth of two *S. rugosoannulata* strains under low-temperature stress

**Fig. 1:**
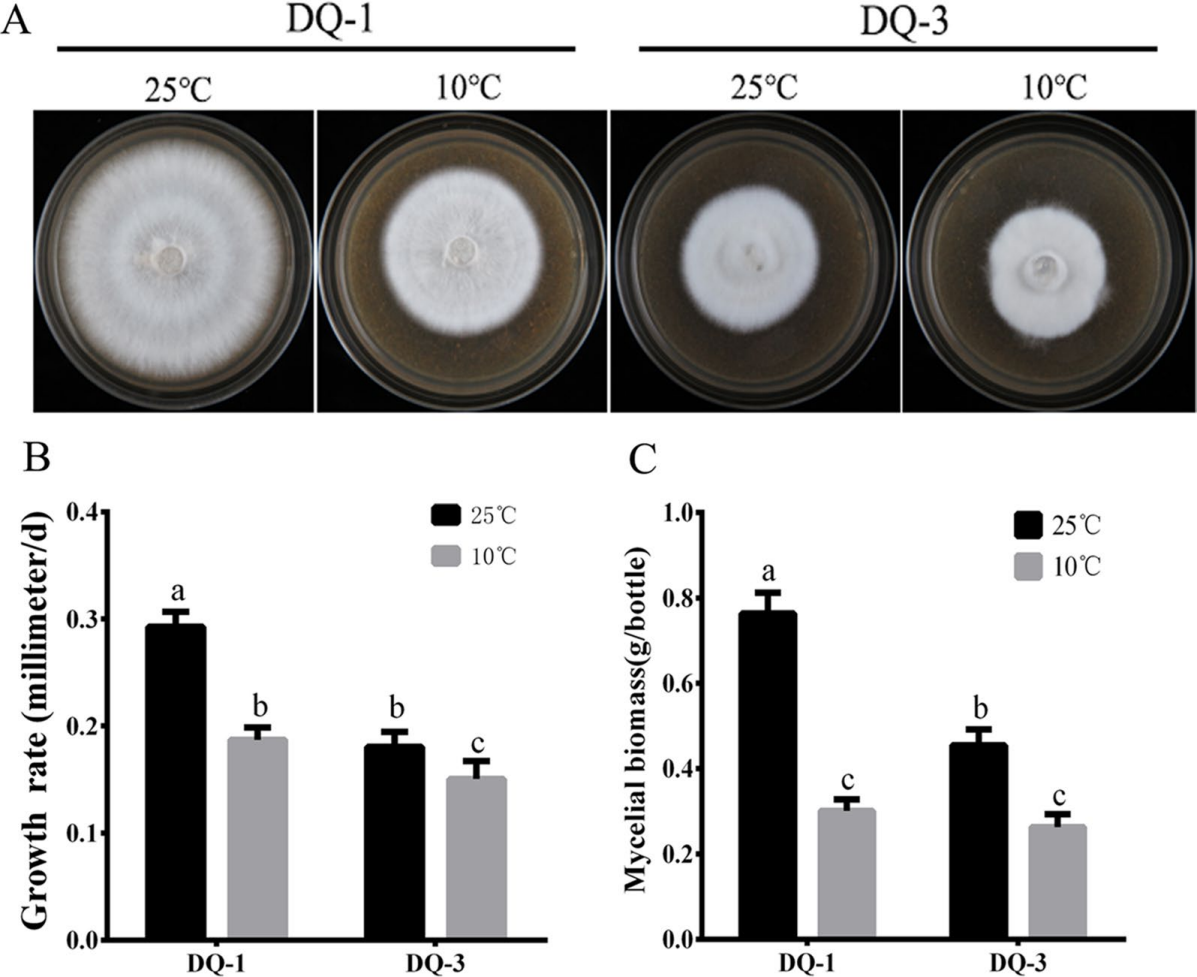
Physiological performance of the *S. rugosoannulata* DQ-1 and DQ-3 strains under the different temperature treatments. **A** and **B** Mycelial growth state and growth rate of the DQ-1 and DQ-3 strains after treatment at 25 °C and 10 °C. **C** Mycelial biomass of strains DQ-1 and DQ-3 after treatment at 25 °C and 10 °C. The error bars represent the means ± standard deviations of triplicate experiments

To test the performance of the DQ-1 and DQ-3 strains under low-temperature stress, plates and liquid mycelia of the two strains were placed at 10 °C for 3 days and mycelia grown at 25 °C were used as controls. As shown in Fig. 1, we found that the growth rate of both *S. rugosoannulata* strains decreased under low-temperature stress (Fig. 1A). The growth rate of the DQ-1 strain dropped from 0.293 ± 0.014 to 0.187 ± 0.012 mm/d, while that of DQ-3 dropped from 0.181 ± 0.013 to 0.150 ± 0.017 mm/d (Fig. 1B). In addition, the dry weight of the mycelia of the DQ-1 strain decreased by 60.63% and that of the DQ-3 strain decreased by 42.21% after low-temperature stress. However, the difference in the dry weight of mycelia between DQ-1 and DQ-3 was not obvious under low-temperature stress (Fig. 1C). Therefore, the growth rate of DQ-1 mycelia and the decrease in biomass were significantly higher than those of DQ-3 under low-temperature stress. Moreover, these findings indicate that the DQ-1 strain is high-sensitive to low temperature while the DQ-3 strain is low-sensitive.

### Transcriptome sequencing, gene mapping and differential expression analysis

**Fig. 2:**
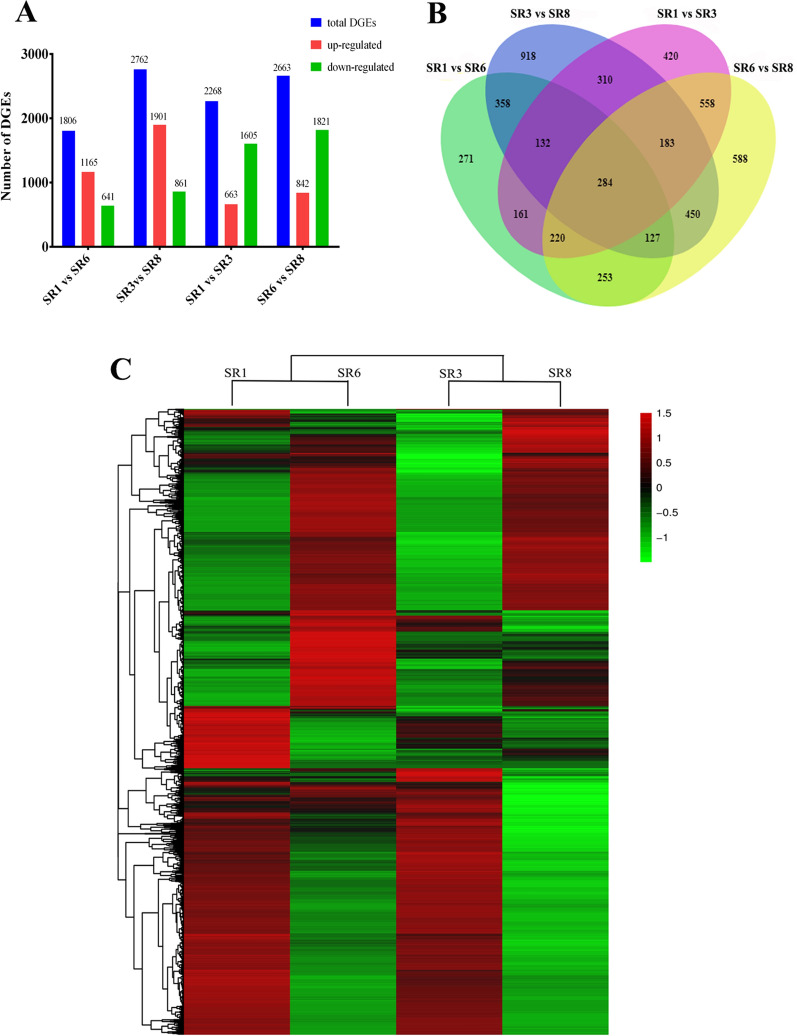
Statistic of different expressed genes. **A** Number of differentially expressed genes (DEGs) after the temperature treatments. **B** Venn diagram of DEGs among the different comparisons. **C** Heat map showing the expression level of DEGs

RNA-Seq was performed using 12 *S. rugosoannulata* cDNA libraries. A total of 592.79 million raw reads were generated by Illumina sequencing. Then, after applying cleaning and quality control, 525.63 million clean reads were obtained, and the Q30 value of the base ratio was higher than 92.26%. The proportion of reads mapping to the *S. rugosoannulata* genome was 95.56–96.89% (Additional file 1: Table S2). Up to 9646 genes and 11,496 transcripts were identified. The expression analysis of SR1 vs. SR6, SR3 vs. SR8, SR1 vs. SR3 and SR6 vs. SR8 generated 9499 DEGs. The partitioning of this value is shown in Fig. 2A. Compared with the DQ-1 strain, DQ-3 had more DEGs after low-temperature stress and showed a higher number of upregulated and downregulated genes. More DEGs were produced between the two strains after low-temperature stress (Fig. 2B). In addition, compared with the DQ-3 strain, the DQ-1 strain had more downregulated genes.

A Venn diagram comparison among DEGs is shown in Fig. 2B, and it revealed that 284 DEGs overlap among the experimental groups. After 10 °C low-temperature treatment, 774 DEGs overlapped between SR1 vs. SR6 and SR3 vs. SR8. After the two varieties were treated with varying temperatures, 1025 DEGs overlapped between SR1 vs. SR3 and SR6 vs. SR8. Moreover, SR3 vs. SR8 had the most unique DEGs. In addition, the DQ-1 and DQ-3 strains had the same overall change trend of differentially expressed genes after low-temperature stress. However, differences in gene expression were still observed between them (Fig. 2C). This result suggests that these differentially expressed genes may be caused by the different responses of the two strains to low temperature.

### GO and KEGG enrichment analyses of DEGs between DQ-1 and DQ-3

**Fig. 3:**
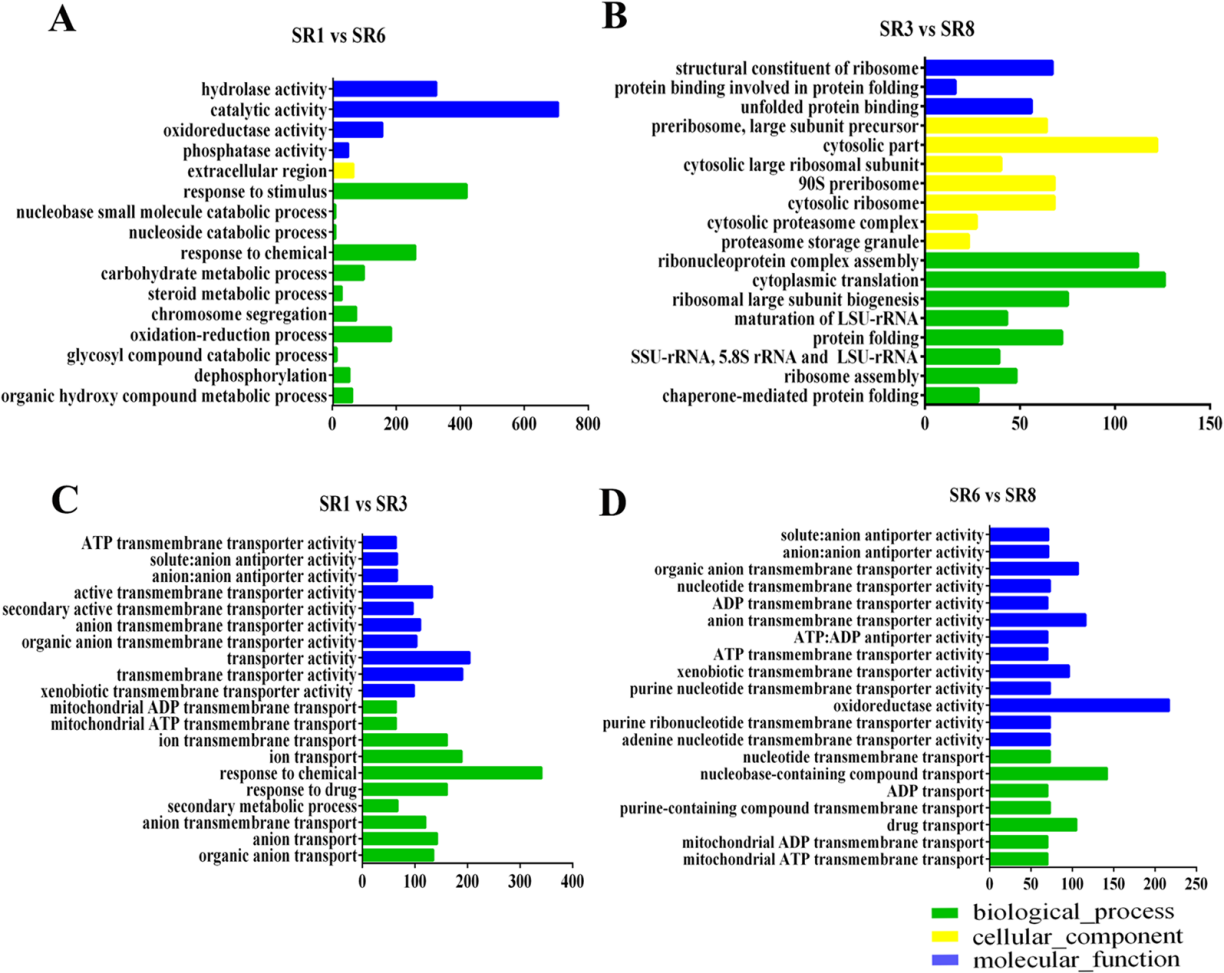
GO functional classification of differentially expressed genes. **A**–**C** and **D** GO enrichment between different treatments. The green bars represent biological processes; yellow bars represent cellular components; and blue bars represent molecular functions. Only the significant GO terms (P < 0.005) were shown

Gene Ontology and KEGG pathway enrichment analyses of DEGs were performed to investigate the effect of low-temperature stress on the up- and downregulation of genes. The number of DEGs upregulated by the two strains increased significantly after low-temperature stress. Based on the GO enrichment analysis, the sample DEGs were assigned to three categories (biological process, cellular component, and molecular function). After the same variety was treated at 10 °C, DQ-1 corresponded to more gene processes, including “catalytic activity”, “hydrolase activity”, “oxidoreductase activity”, “response to stimulus”, “response to chemical”, “oxidation–reduction process” and “extracellular region” (Fig. 3A). However, DQ-3 mainly corresponded to “structural constituent of ribosome”, “unfolded protein binding”, “cytosolic part”, “cytosolic ribosome”, “ribonucleoprotein complex assembly” and “cytoplasmic translation” (Fig. 3B). After treating the different strains at 25 °C, more genes were involved in “transporter activity”, “transmembrane transporter activity”, “active transmembrane transporter activity”, “response to chemical” and “anion transport” (Fig. 3C), whereas after treatment at 10 °C, these genes were mostly involved in “oxidoreductase activity”, “anion transmembrane transport” and “nucleobase-containing compound transport” (Fig. 3D). However, none of them involved cellular components, and a more detailed GO enrichment process is shown in Additional file 1: Table S3.

The DEGs from SR1 vs. SR6, SR3 vs. SR8, SR1 vs. SR3 and SR6 vs. SR8 revealed 10, 6, 11 and 8 significantly enriched pathways (P < 0.01), respectively (Additional file 1: Table S4). After the same variety was treated at 10 °C, the pathways enriched by the DQ-1 strain included “microbial metabolism in diverse environments”, “aminobenzoate degradation”, “fatty acid degradation” and “pentose and glucuronate interconversions”, while those enriched by the DQ-3 strain included “proteasome”, “ribosome”, “ribosome biogenesis in eukaryotes” and “steroid biosynthesis”. At the same time, the “steroid biosynthesis” pathway was shared by the two strains after low-temperature stress (Additional file 1: Fig. S1). After the different strains were treated at 25 °C, the DEG enrichment pathways included “microbial metabolism in diverse environments”, “glycine, serine and threonine metabolism”, “degradation of aromatic compounds” and “carbon metabolism”. However, low-temperature treatment at 10 °C induced some specific enriched significant enrichment pathways (steroid biosynthesis and unsaturated fatty acid biosynthesis) (Additional file 1: Table S4).

The results of the GO and KEGG functional classification and enrichment analyses indicated that there were certain differences between the two strains of *S. rugosoannulata* after low-temperature stress. However, they were more involved in certain processes, such as xenobiotic biodegradation and metabolism, carbohydrate metabolism, lipid metabolism and oxidoreductase activity. Thus, these processes may play an important role in resisting low-temperature stress.

### Differential expression of CAZyme genes and analysis of cellulase activity in *S. rugosoannulata* under low-temperature stress

**Fig. 4:**
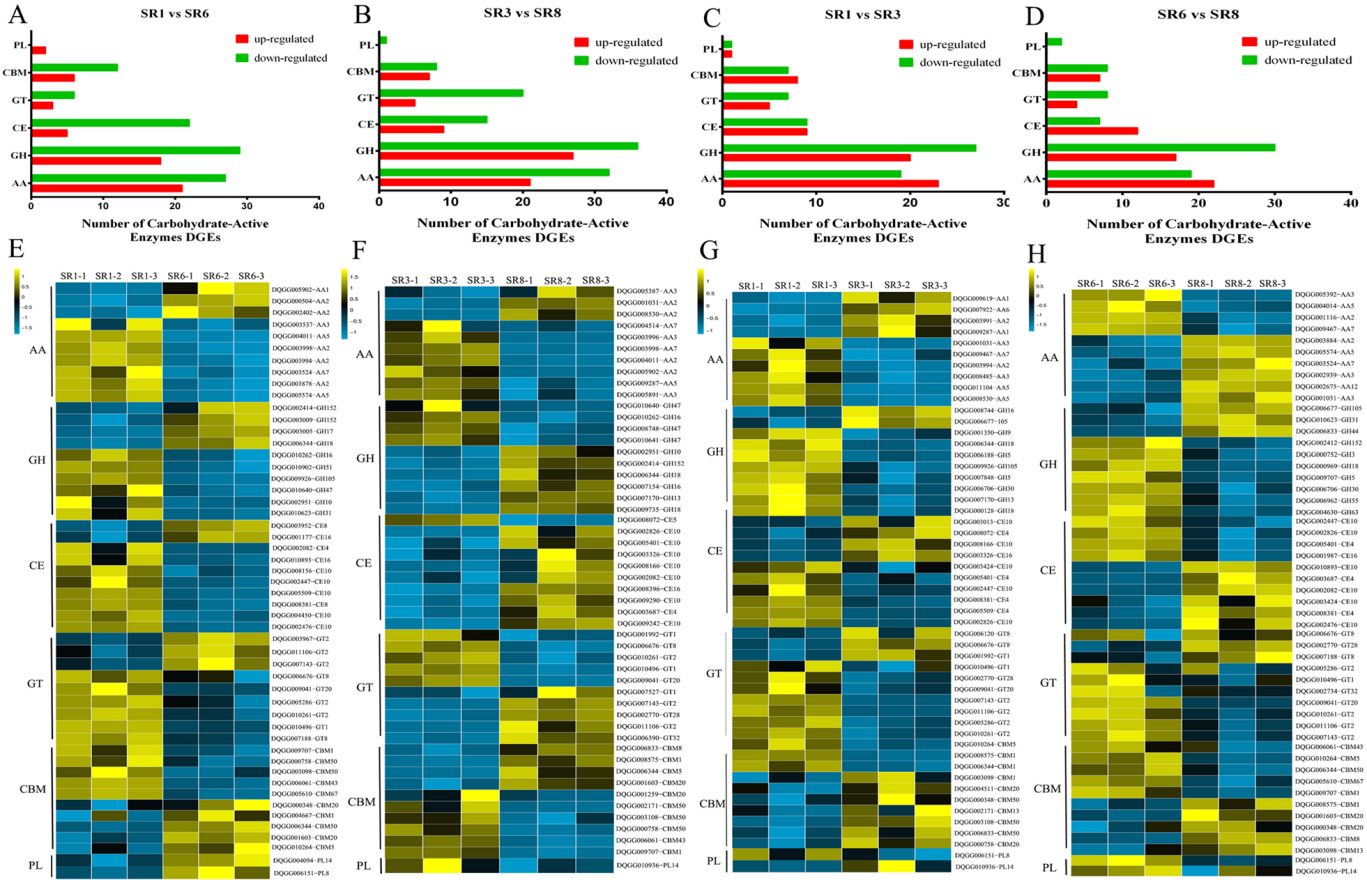
Difference analysis of carbohydrate enzyme genes under the different temperature treatments. **A**–**D** Comparison of the number of differentially expressed carbohydrate enzyme genes between different treatments. **E–H** Expression levels of the top ten genes with significant changes in each carbohydrate enzyme (AA, GH, CE, GT, CBM and PL) family under the different treatments

Carbohydrate enzymes are the key enzymes used by fungi for nutrient transformation and utilization and mainly include six families of enzymes (auxiliary activities (AAs), glycosyl hydrolases (GHs), carbohydrate binding modules (CBMs), carbohydrate esterases (CEs), glycosyl transferases (GTs), and polysaccharide lyases (PLs)) (Hao et al. 2019). By comparing the whole transcriptome of *S. rugosoannulata* with the CAZy database, we identified 181 DEGs as CAZyme genes. After the two strains were subjected to low-temperature stress, we found that their carbohydrate enzyme expression patterns had more downregulated genes, with the DQ-3 strain showing more DEGs (Fig. 4A, B). At the same time, the top ten gene cluster analyses for six carbohydrate enzymes with significant differences were selected, as shown in Fig. 4. We found that the DQ-1 strain showed greater downregulation of AA, GH, CE, and GT family genes, while the DQ-3 strain showed greater upregulation of the GH, CE, and GT family genes (Fig. 4E, F). In addition, when treated at 25 ℃ and 10 ℃, the GH family genes of the DQ-3 strain were downregulated considerably, while other enzyme families did not change much (Fig. 4C, D). In addition, after culturing the two strains at 25 °C, the AA, GH, CE and GT genes of the DQ-3 strain were significantly downregulated (Fig. 4G). However, certain carbohydrate-enzyme genes were upregulated under 10 °C low-temperature stress (Fig. 4H).

**Fig. 5:**
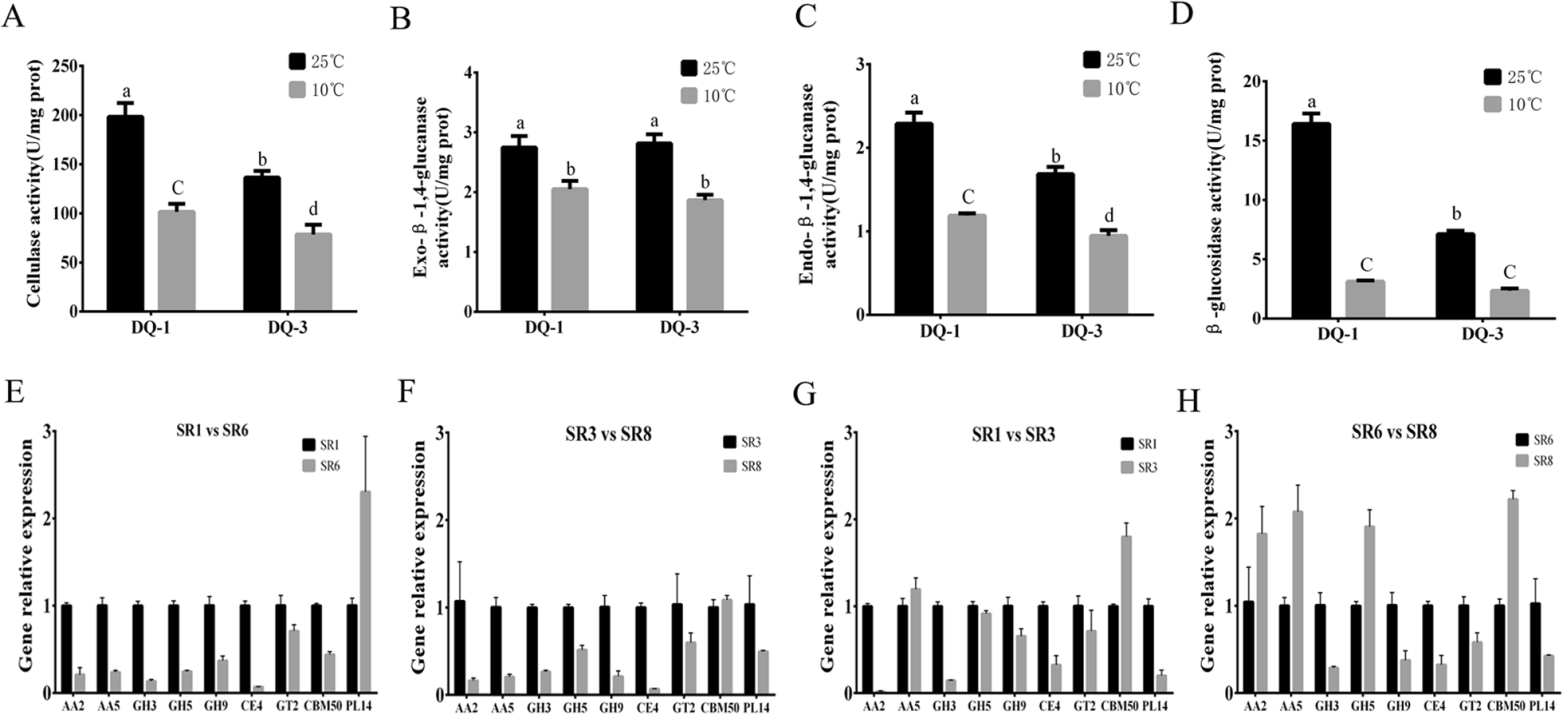
Analysis of carbohydrate enzyme activity and gene expression level verification under the different temperature treatments*.* **A**–**D** Changes in cellulase, exo/endo-β-1,4-glucanase and β-glucosidase activity under the different treatments. **E**, **F** Validation of the expression levels of nine differentially expressed CAZymes (AA2, AA5, GH3, GH5, GH9, CE4, GT2, CBM50 and PL14). The error bars represent the means ± standard deviations of triplicate experiments

To further illustrate the effect of low-temperature stress on the carbohydrate metabolism of *S. rugosoannulata*, we measured the activities of four important carbohydrate enzymes (cellulase, exo/endo-β-1,4-glucanase and β-glucosidase) and found that the four enzymes showed a downward trend after low-temperature stress (Fig. 5). In addition, there was no significant change in endo-β-1,4-glucanase activity of the two strains, while the enzymes showed significantly lower activity in the DQ-3 strain than the DQ-1 strain under the 25℃ treatment. After low-temperature stress, the DQ-1 strain showed reductions in cellulase and endo-β-1,4-glucanase activity by 48.24–48.71%, and that of the DQ-3 strain decreased by 42.42–43.50% (Fig. 5A, C). Exo-β-1,4-glucanase activity also decreased by 25.34% and 33.70% in the DQ-1 and DQ-3 strains, respectively (Fig. 5B). In addition, the activity of β-glucosidase decreased most significantly, with the DQ-1 strain showing a decrease of 80.99% and the DQ-3 strain showing a decrease of 67.2% (Fig. 5D).

To confirm the reliability of the carbohydrate enzyme gene expression pattern, the six selected enzyme family genes were validated using qRT–PCR. These genes included 2 AA genes [*AA2* (DQGG003998) and *AA5* (DQGG004011)], 3 GH genes [*GH3* (DQGG000752), *GH5* (DQGG009707) and *GH9* (DQGG002370)], 1 *CE4* (DQGG002082) gene, 1 *GT2* (DQGG005286) gene, 1 *CBM50* (DQGG000758) gene and 1 *PL14* (DQGG010936) gene. As shown in Fig. 5E-F, gene expression profiling of carbohydrate enzymes using qRT–PCR revealed variation trends similar to those observed in the RNA-Seq results. Low-temperature stress has a certain effect on carbohydrate enzymes of *S. rugosoannulata*, and it reduces the process of carbohydrate metabolism by downregulating the expression of AA, GH, CE and GT family genes.

### Differential expression of antioxidant enzyme genes and enzyme activity analysis of *S. rugosoannulata* under low-temperature stress

**Fig. 6:**
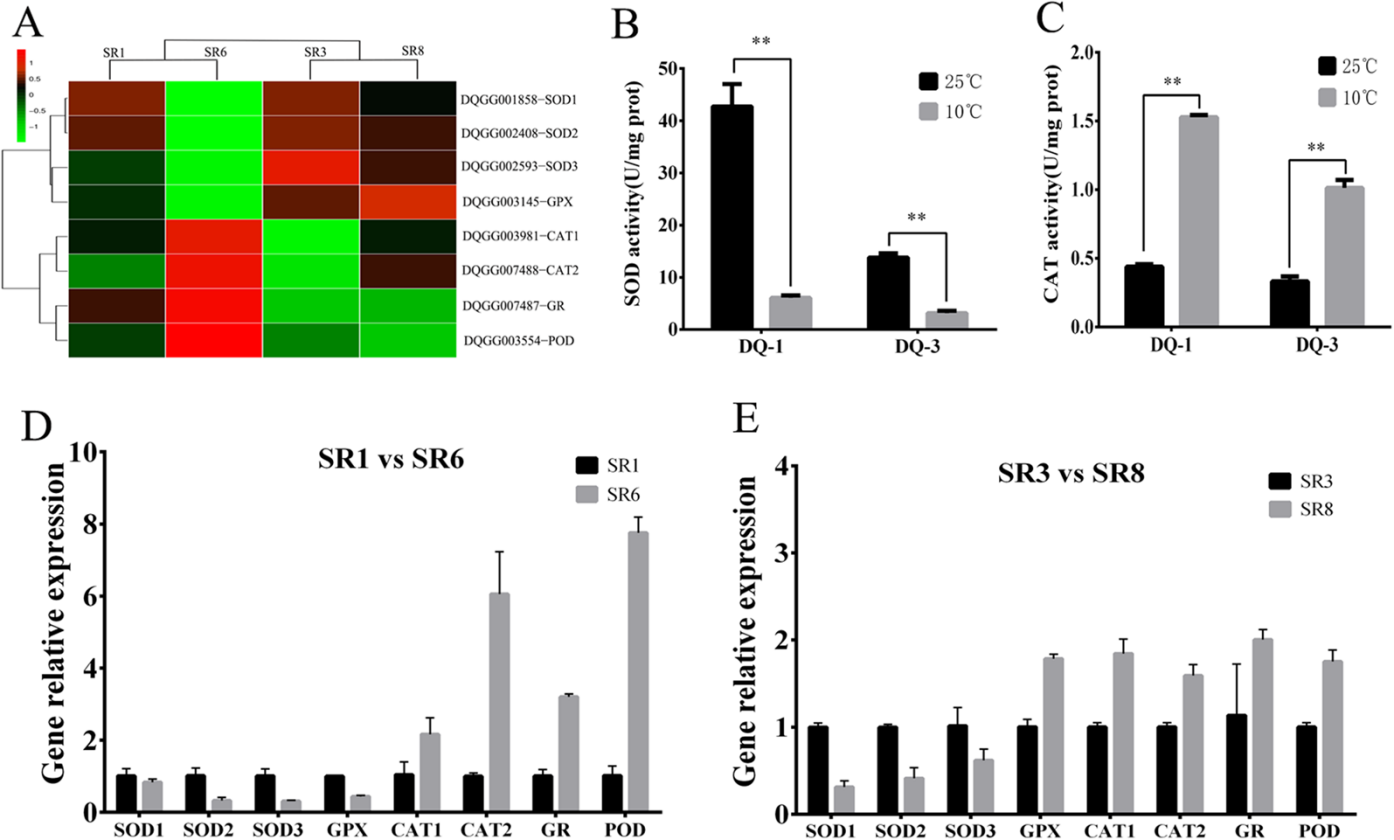
Analysis of antioxidant enzyme activity and gene expression levels under the different temperature treatments. **A** Transcriptome comparative analysis of the expression of antioxidant enzyme genes between different treatments. **B** and **C** Changes in SOD and CAT enzyme activities in the DQ-1 and DQ-3 strains under the different temperature treatments. **D** and Validation of the expression levels of eight differentially expressed antioxidant enzymes (SOD1, SOD2, SOD3, GPX, CAT1, CAT2, GR and POD). The error bars represent the means ± standard deviations of triplicate experiments

Studies have shown that low-temperature stress can cause oxidative stress in fungi, which can resist oxidative damage through antioxidant enzymes (Hu et al. 2009). In the GO enrichment analysis, we also found that the process of “oxidoreductase activity” can be significantly enriched after low-temperature stress; therefore, the differential expression of oxidoreductase may play an important role in resisting low-temperature stress. Therefore, we analyzed the expression of genes with antioxidant enzymes and found that the *SOD1* (DQGG001858), *SOD2* (DQGG002408) and *SOD3* (DQGG002593) genes of the two strains were all downregulated under low-temperature stress. Among them, more significant downregulation was observed for the DQ-1 strain. However, the *CAT1* (DQGG003981), *CAT2* (DQGG007488), glutathione reductase (*GR*, DQGG007487) and peroxidase (*POD*, DQGG003554) genes of the two strains were upregulated after low-temperature stress, and the upregulated expression of the DQ-1 strain was more significant. It is worth noting that the expression level of the glutathione peroxidase (*GPX*, DQGG003145) gene of the DQ-1 strain was downregulated after low-temperature stress, while the expression level of the DQ-3 strain was upregulated (Fig. 6A).

We further determined the SOD and CAT activities of the two strains after low-temperature stress. The SOD enzyme activity of the DQ-1 strain was 3.09 times that of DQ-3 under mycelial culture conditions at 25 °C, whereas the difference in CAT enzyme activity was not obvious. After 10 °C low-temperature stress, the SOD enzyme activity of the DQ-1 and DQ-3 strains decreased by 85.75% and 77.08%, respectively (Fig. 6B, C). However, the CAT enzyme activity of the DQ-1 and DQ-3 strains increased by 3.45 times and 3.06 times, respectively (Fig. 6B, C). At the same time, we also verified the expression of all antioxidant enzyme genes through qRT–PCR, which is consistent with the RNA-Seq and enzyme activity results (Fig. 6D, E). This result indicates that *S. rugosoannulata* might resist low-temperature stress by activating *CAT*, *GR* and *POD* and the DQ-3 strain may also upregulate *GPX* gene expression to increase its tolerance to low-temperature stress.

### Heat shock proteins of *S. rugosoannulata* in response to low-temperature stress

**Fig. 7:**
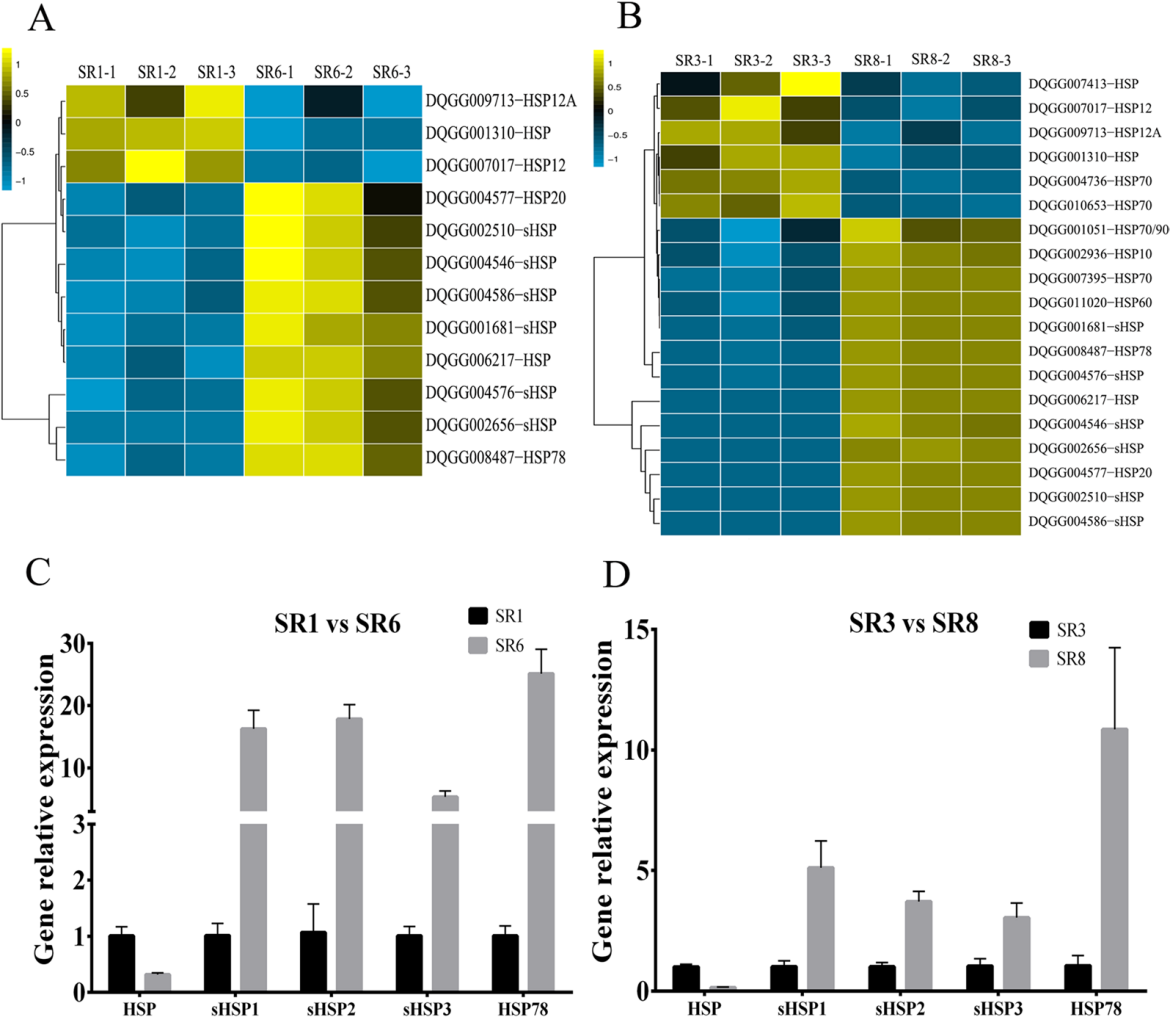
Analysis of heat shock protein expression under the different temperature treatments. **A** and **B** Transcriptome comparative analysis of the expression of heat shock protein genes between different treatments. **C** and **D** Validation of the expression levels of five differentially expressed heat shock proteins (HSP, sHSP1, sHSP2, sHSP3 and HSP78). The error bars represent the means ± standard deviations of triplicate experiments

Heat shock proteins (HSPs) are proteins synthesized by biological cells to resist high-temperature stress. However, studies have also shown that heat shock proteins can maintain the normal structure of functional proteins, prevent protein degeneration, enhance cell membrane fluidity and maintain the normal physiological functions of cells under low-temperature stress (Renaut et al. 2006). After 10 °C low-temperature stress, the DQ-1 strain had 3 downregulated HSP expression levels and 9 upregulated HSP gene expression levels. In addition, the DQ-3 strain had 6 downregulated HSP expression levels and 13 upregulated HSP gene expression levels (Fig. 7A, B). It is worth noting that the expression of 6 small HSPs was significantly upregulated in both strains. In addition, we selected 1 *HSP* (DQGG006217), 3 sHSPs [*sHSP1* (DQGG002510), *sHSP2* (DQGG002656) and *sHSP3* (DQGG004546)] and 1 *HSP78* (DQGG008487) for the qRT–PCR verification experiments, which is consistent with the RNA-Seq results (Fig. 7A, B). This result indicates that HSPs may play an important role in resisting low-temperature stress in *S. rugosoannulata*. In addition, the expression of more HSP genes in the DQ-3 strain was upregulated, which may be related to low-temperature tolerance.

## Discussion

*S. rugosoannulata* is an edible and medicinal fungus (Huang et al. 2010). In recent years, due to its easy cultivation, high yield and rough management, *S. rugosoannulata* cultivation on forestland and fields has become popular in China (Gong et al. 2018). However, *S. rugosoannulata* can only be cultivated according to seasonal temperature changes and will experience cold winters (Ren et al. 2020). Therefore, the low-temperature environment during the overwintering process will not only reduce the mycelial metabolic activity and affect the growth of the mycelia but also cause stress damage to the mycelia. To breed varieties with greater resistance to low-temperature stress and higher degradation efficiency, we used transcriptomics to study two strains with different temperature sensitivities and degradation efficiencies to better understand the response mechanism of *S. rugosoannulata* under low temperatures.

Low temperature has a certain inhibitory effect on the growth of fungi, and it is also used as an important method of regulating growth and development (Sakamoto, 2018). However, differences are observed in the sensitivity of different types or varieties of edible fungi to temperature. In the present study, through preliminary screening experiments on the collected seed resources, we found that DQ-1 mycelium grew faster than DQ-3 mycelium, although the mycelial growth rate and biomass also decreased more after low-temperature treatment (Fig. 1). Therefore, we determined that the DQ-1 strain is more sensitive to low temperature while the DQ-3 strain is less sensitive. Furthermore, we performed transcriptome sequencing of these two strains under low-temperature treatment and obtained a total of 525.63 million clean reads. In addition, the reads of these samples could be mapped to the *S. rugosoannulata* genome at a rate of 95.56–96.89% (Additional file 1: Table S2). In addition, we identified 9646 genes and 11,496 transcripts. However, the transcriptome analysis of *P. pulmonarius* after the low-temperature treatment showed that only 82.59–83.22% of the transcriptome mapped to the reference genome (Wang et al. 2020). Thus, our results provide more comprehensive information.

The comparative transcriptome analysis revealed that many more DEGs were activated in response to low-temperature stress in the DQ-3 strain than the DQ-1 strain. Moreover, 1901 DEGs were upregulated in the DQ-3 strain after low-temperature stress while only 1165 DEGs were upregulated in the DQ-1 strain (Fig. 2A). The differentially expressed genes of *P. pulmonarius* also increased with the extension of the low-temperature treatment time, and the upregulated DEGs accounted for 53% of the genes after 12 h of treatment (Xie et al. 2018). This result may also indicate that more DEGs of DQ-3 are related to resistance to cold stress. The DQ-1 strain had more downregulated DEG genes than the DQ-3 strain (Fig. 2B), which indicates that DQ-3 may have a more complex gene regulatory network to respond to changes in environmental factors. However, the overall expression trends of DEG genes after the low-temperature treatment of the two strains were basically similar (Fig. 2C). Therefore, the mechanisms of different varieties of *S. rugosoannulata* in response to low-temperature stress should be conservative.

GO and KEGG enrichment analyses have been used as routine methods to analyze the transcriptome of many edible fungi, such as *Hypsizigus marmoreus* (Zhang et al. 2015), *Morchella importuna* (Hao et al. 2019) and *V. volvacea* (Bao et al. 2013). Our results show that after the temperature drops, the GO terms of the DQ-1 strain mainly enrich hydrolase activity, oxidoreductase activity, and stimulus responses. However, the DQ-3 strain mainly involves the structural constituents of ribosomes, unfolded protein binding, cytosolic parts, and cytosolic ribosomes (Fig. 3). A study of the resistance of peach and *Malus baccata* to low-temperature stress showed that DEGs are involved in hydrolase activity, temperature stimulus, oxidoreductase activity and protein binding (Jiao et al. 2016; Li et al. 2018). Under different stress conditions, *Arabidopsis* is also involved in the structural constituents of ribosomes, unfolded protein binding and cytosolic ribosomes (Jin et al. 2020; Deng et al. 2011; Beine-Golovchuk et al. 2018). These processes are similar to those involved after low-temperature stress in *S. rugosoannulata* and may be essential for *S. rugosoannulata* to respond to low-temperature stress. In addition, the KEGG enrichment of these two strains mainly identified xenobiotic biodegradation and metabolism, carbohydrate metabolism, and lipid metabolism pathways (Additional file 1: Table S4). This result indicates that low temperature has an important influence on the metabolic activities of *S. rugosoannulata*, especially the process of carbohydrate metabolism, and it also implies that *S. rugosoannulata* may improve its cold tolerance through oxidoreductase activity, lipid accumulation, protein folding or ribosome remodeling.

In *Agaricus bisporus*, carbohydrate enzymes are important enzymes that degrade lignocellulose in straw (Morin et al. 2012). *S. rugosoannulata* and *A. bisporus* are both white rot fungi and have a rich variety of carbohydrate enzymes (Huang et al. 2010). In our research, a total of 181 DEGs were identified as carbohydrate enzymes. After the low-temperature treatment, more carbohydrate enzyme genes of the DQ-3 strain were downregulated (Fig. 4A, B). At the same time, we focused on analyzing the top ten carbohydrate enzyme DEG genes and found that they mainly involve AA, GH, CE, and GT family genes (Fig. 4). Furthermore, we measured the cellulase, exo-β-1,4-glucanase (GH5), endo-β-1,4-glucanase (GH9) and β-glucosidase enzyme (GH3) activities and found that low temperature caused a significant decrease in enzyme activity, with a greater decrease observed in the DQ-1 strain (Fig. 5). Therefore, the cellulase genes belonging to these GH families are greatly affected by low temperature. Previous studies have confirmed that the GH, CE, and PL families are involved in the process of fungal degradation of plant cell walls (Ospina-Giraldo et al. 2010). Additionally, a transcriptomics analysis of low-temperature-tolerant varieties revealed the differential expression of 18 PLs in *V. volvacea* (Bao et al. 2013); however, we only identified 2 PL genes in *S. rugosoannulata*. These results indicate that different fungi have different types of carbohydrate enzyme degradation substrates. However, few studies have focused on the expression of fungal carbohydrate enzyme genes at low temperatures. Therefore, our research on the differential expression of carbohydrate enzyme genes under low-temperature conditions will provide a useful reference for similar studies on other fungi.

Low temperature is one of the main abiotic stress factors, and it can be divided into cold stress (0–15 ℃) and freezing stress (< 0℃) (Guo et al. 2018). After mycelia were exposed to low-temperature stress at 10 °C, we found that the expression of the antioxidant enzyme genes *SOD1*, *SOD2*, and *SOD3* was downregulated while the expression of the genes *CAT1*, *CAT2*, *GR* and *POD* was upregulated (Fig. 6A). In addition, the DQ-1 strain showed higher upregulation than the DQ-3 strain. It is worth noting that the expression of the *GPX* gene was downregulated in DQ-1 after low-temperature stress but upregulated in DQ-3. Furthermore, we studied the enzyme activity of SOD and CAT and found that it was consistent with the gene expression (Fig. 6B, C). Previous studies have shown that plants can resist low-temperature stress through antioxidant enzymes, and it has been found that the enzyme activity of cold-sensitive plants (such as cucumber and maize) decreases more significantly (Lukatkin 2002). The study also found that tomato can increase the activity of POD and CAT through the transcription factor *ICE*_*1*_ to improve cold resistance (Yu et al. 2015). This finding is similar to the results of our study on the low-temperature activation of CAT and POD gene expression, which improved cold tolerance. In edible fungi, the SOD, CAT and POD activity of *V. volvacea* initially increased and then decreased after 2–8 h of low-temperature stress. However, the antioxidant enzyme (SOD, CAT and POD) activity of *H. marmoreus* increased after low-temperature stress (Hu et al. 2009). In addition, *H. marmoreus* can also use antioxidant enzymes (GPX and GR) to remove ROS caused by mechanical damage (Chen et al. 2018). In our research, DQ-3 may also use GPX and GR to reduce oxidative stress caused by low temperature. This result indicates that *S. rugosoannulata* may resist oxidative damage caused by low temperature through antioxidant enzymes (CAT, GPX, GR and POD), and there are certain differences between different varieties.

At present, the expression of heat shock proteins in plants is considered diverse, and it occurs under heat stimulation and cold stress, during seed germination and during maturation (Yang et al. 2007). Moreover, heat shock proteins may function as molecular chaperones to help refold the denatured proteins caused by cold stress (Yang et al. 2007). Collins et al. (1995) first confirmed the direct relationship between HSP and cold tolerance and found that HSP70 and HSP79 reduced cold stress damage to plant cell membranes, thereby increasing the plant’s cold tolerance. Our research found that the expression of a small number of HSP genes was downregulated in the two strains after low-temperature stress while most of the genes were upregulated (Fig. 7). In addition, the DQ-3 strain showed greater upregulation of the HSP gene, which may be the reason why DQ-3 has stronger low-temperature resistance. These upregulated genes mainly include *HSP20*, *HSP78*, *HSP60*, *HSP70*, *HSP90* and *sHSP*. It is worth noting that the DQ-1 and DQ-3 strains each showed upregulated expression of 6 sHSP genes. In edible fungi, *HSP90* can enhance the tolerance of *V. volvacea* to low temperature (Huang et al. 2017). In the yeast *S. cerevisiae*, the stress-induced increase in *sHSP* gene transcription is also very high and *sHSP* usually represents one of the most upregulated stress-related genes (Solís et al. 2016; Georg et al. 2020). This finding is consistent with our results showing that the *sHSP* gene in the HSPs is upregulated the most under low-temperature stress. In addition, the chaperone family of small heat shock proteins can cope with cell function damage and protein misfolding caused by various stresses (Haslbeck et al. 2015). Therefore, this result suggests that *S. rugosoannulata* may use heat shock proteins to resist cold stress, with *sHSP*, *HSP20* and *HSP78* playing important roles. In addition, the expression of more HSP genes may also be a sign of more low-temperature tolerance in the DQ-3 strain.

In summary, transcriptome sequencing technology was used to clarify the gene expression changes, the type and number of differentially expressed genes, GO classification and metabolism process between the DQ-1 strain and DQ-3 strain of *S. rugosoannulata* under low-temperature stress. This research found that the two *S. rugosoannulata* strains presented greater differential expression of the AA, GH, CE, and GT family genes, with the DQ-1 strain showing greater downregulated gene expression. Further study of the antioxidant enzyme genes of the two strains found that *CAT1*, *CAT2*, *GR*, and *POD* were all upregulated, although the differential expression of the antioxidant enzyme genes of the DQ-1 strain was more obvious. It is worth noting that *GPX* is only upregulated in DQ-3. In addition, both strains showed higher upregulation of heat shock proteins, with DQ-3 presenting a higher number of upregulated genes. This result shows that low temperature can reduce the metabolism of different types of carbohydrate enzymes to affect mycelial growth and can enhance the cold-tolerance characteristics of *S. rugosoannulata* through different antioxidant enzymes and heat shock proteins. The results of this study provide scientific data for further studying the expression patterns of *S. rugosoannulata* cold tolerance genes, exploring the regulatory relationship between differentially expressed genes and mycelial cold tolerance, and revealing the relevant cold tolerance mechanisms of *S. rugosoannulata*.

## Supplementary Information

**Additional file 1:** **Figure S1.** KEGG enrichment analysis of DEGs under different temperature treatments. **Table S1.** Primers for qRT-PCR of the validation gene. **Table S2.** Summary of the sequencing data of *Stropharia rugosoannulata* transcriptome at different temperature treatments. **Table S3.** GO functional classification of differentially expressed genes. **Table S4.** KEGG pathways enrichment analysis of differentially expressed genes.

## Data Availability

Data supporting the conclusions are presented in the main article.

## Abbreviations

GO: Gene Ontology function database
KEGG: Kyoto Encyclopedia of Genes and Genomes pathway database
DEG: Differentially expressed gene
CAZyme: Carbohydrate active enzymes
qRT–PCR: Quantitative real-time PCR
AAs: Auxiliary activities
GHs: Glycosyl hydrolases
CBMs: Carbohydrate binding modules
CEs: Carbohydrate esterases
GTs: Glycosyl transferases
PLs: Polysaccharide lyases
SOD: Superoxide dismutase
CAT: Catalase
HSP: Heat shock protein
GPX: Glutathione peroxidase
GR: Glutathione reductase
POD: Peroxidase
APX: Ascorbate peroxidase
ROS: Reactive oxygen species
