# Correction: Transcriptomic analysis of *Stropharia rugosoannulata* reveals carbohydrate metabolism and cold resistance mechanisms under low-temperature stress

**DOI:** 10.1186/s13568-022-01412-y

**Published:** 2022-06-13

**Authors:** Haibo Hao, Jinjing Zhang, Shengdong Wu, Jing Bai, Xinyi Zhuo, Jiaxin Zhang, Benke Kuai, Hui Chen

**Affiliations:** 1 State Key Laboratory of Genetic Engineering and Fudan Center for Genetic Diversity and Designing Agriculture, Institute of Plant Biology, School of Life Sciences, Fudan University, 413 Room, No. 2005, Songhu Road, YangPu District, Shanghai, 200438 China; 2 National Research Center for Edible Fungi Biotechnology and Engineering, Key Laboratory of Applied Mycological Resources and Utilization, Ministry of Agriculture, Shanghai Key Laboratory of Agricultural Genetics and Breeding, Institute of Edible Fungi, Shanghai Academy of Agricultural Sciences, 309 Room, No. 1000, Jinqi Road, FengXian District, Shanghai, 201403 China

## Correction to: AMB Express (2022) 12(1): 1–5 https://doi.org/10.1186/s13568-022-01400-2

Following publication of the original article (Hao et al. 2022), the authors reported an error in the author group. The typesetter has inadvertently missed to include the dagger symbol for first author. Now this has been corrected with this erratum.

The original article (Hao et al. 2022) has been updated.
