# Supplementary material for: Transcriptomic analysis of Stropharia rugosoannulata reveals carbohydrate metabolism and cold resistance mechanisms under low-temperature stress
Source: AMB Express. 2022 May 14;12:56. doi: 10.1186/s13568-022-01400-2 (PMC9107548; doi:10.1186/s13568-022-01400-2)
Supplement: Supplementary file 1 — Additional file 1: Figure S1. KEGG enrichment analysis of DEGs under different temperature treatments. Table S1. Primers for qRT-PCR of the validation gene. Table S2. Summary of the sequencing data of Stropharia rugosoannulata transcriptome at different temperature treatments. Table S3. GO functional classification of differentially expressed genes. Table S4. KEGG pathways enrichment analysis of differentially expressed genes. [file 13568_2022_1400_MOESM1_ESM.docx]

**Supporting Information**

Figure S1 KEGG enrichment analysis of DEGs under different temperature treatments

Table S1 Primers for qRT-PCR of the validation gene

Table S2 Summary of the sequencing data of *Stropharia rugosoannulata* transcriptome at different temperature treatments.

Table S3 GO functional classification of differentially expressed genes.

Table S4 KEGG pathways enrichment analysis of differentially expressed genes.

Figure S1 KEGG enrichment analysis of DEGs under different temperature treatments


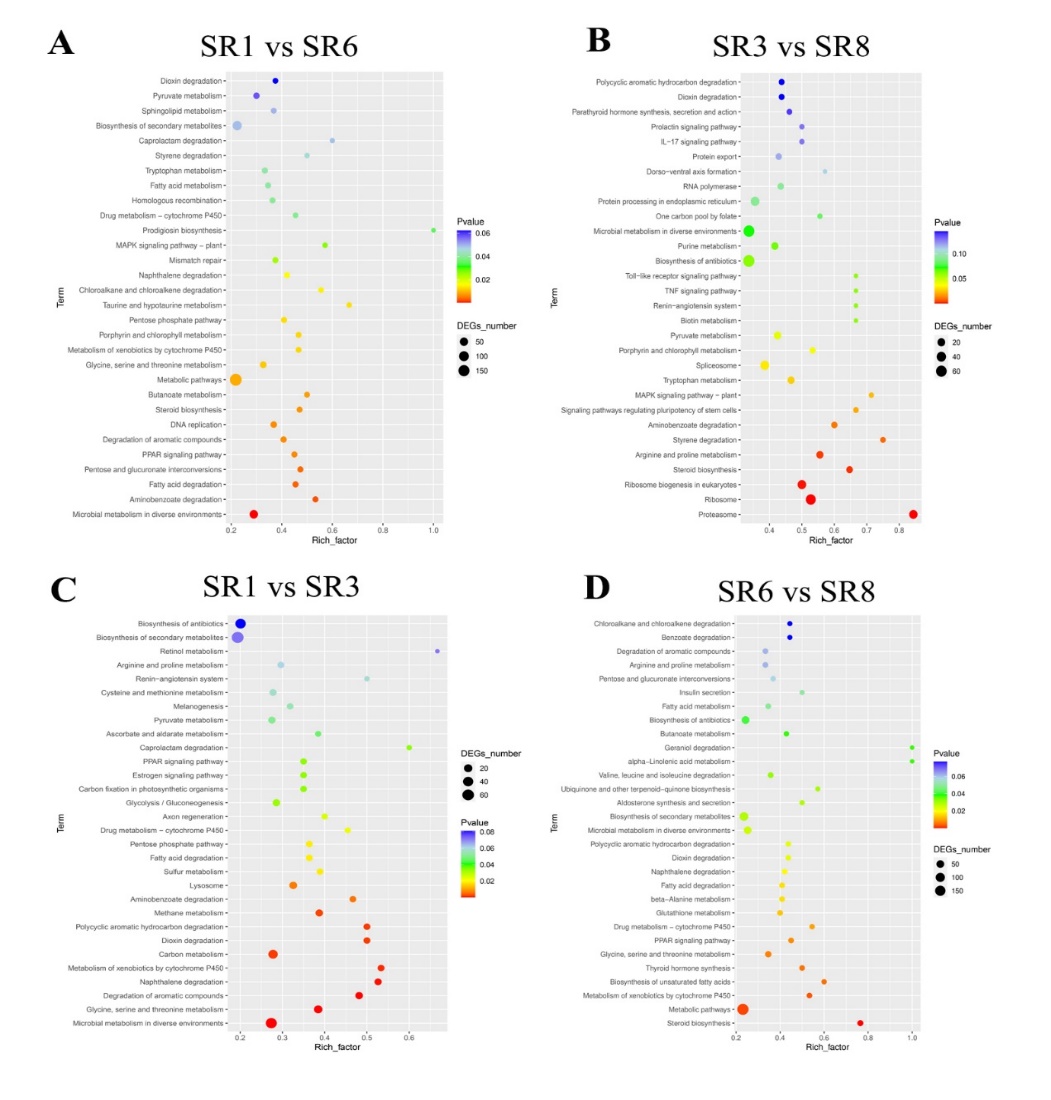


Table S1 Primers for qRT-PCR of the validation gene

| Gene Name | Gene ID | Primer-F | Primer-R |
| --- | --- | --- | --- |
| AA2 | DQGG003998 | ACTCGTGCTCTCTTTGCCTC | GTGGAAGACCAGACGGAGTG |
| AA5 | DQGG004011 | GCGTGAGAGTAACCAACCCA | GACTGGTAGTGGTTCCACCG |
| GH3 | DQGG000752 | CCAGCTCTGGTGTCCAATGT | GTAGGCGTTTCCCATTTCGC |
| GH5 | DQGG009707 | GCTAAATTCGCGTCCAACCC | TTGGAGACGAAGGTCTGTGC |
| GH9 | DQGG002370 | AGATGGATGTCTGGGAGGCT | GGAGTCGTACCTGTTGGTCG |
| CE4 | DQGG002082 | TGCGAATGGGACCTACAACC | AGAAGTTGTGAGCACGGGAG |
| GT2 | DQGG005286 | CTGGTGCTGATCCTGAGCAA | GGAAGGCACGTACGACAGAA |
| CBM50 | DQGG000758 | AGGTCGGAGAGGTCCTTTGT | TTTCCGCATGTCGCATTCAC |
| PL14 | DQGG010936 | GTGTCGATTTGACCACTGCG | CTGTCGCCTCCGTAGAATCC |
| SOD1 | DQGG001858 | GAAAACGCACGGTTCTCCAG | CCGTTCCAGAGTGTACGACC |
| SOD2 | DQGG002408 | CCTCCAGGCAAGCTACTGTC | TACTTCGTTGCCTCCAGCAG |
| SOD3 | DQGG002593 | GGCTCCCTCGACAACTTGAA | GCTCCCAGATATCGACACCG |
| GPX | DQGG003145 | TCAATTCGGTGGTCAGGAGC | GACCGAGAAGACCAGACTCG |
| CAT1 | DQGG003981 | AACTGGGAATCGCTGCCTAC | CGCGCTGCTTTATTCCTGAC |
| CAT2 | DQGG007488 | ATTGTTGTCGTCAAGCACGC | TGTGGCTCCTGCAGAGATTG |
| GR | DQGG007487 | ACGTTTCTGGCTCTCTGTGG | GCGCTGCCCTAAACAGAGTA |
| POD | DQGG003554 | AAGGCTCGCACTTAGTGACC | TAAGCCGAGCCACCTCAAAG |
| HSP | DQGG006217 | TGAACGAAGTTCCCGAAGGG | GCAAGGCGATCGTTGACTTC |
| sHSP1 | DQGG002510 | TGCGCTGGACATCGAGAAAT | AGGCGACCAGGAACCTATCT |
| sHSP2 | DQGG002656 | CCAAGTTCAGAGCCAGAGGG | AAGCTCAAAGGAAGCGGTGA |
| sHSP3 | DQGG004546 | TGTGCACAATAACCGAGGCT | CCGCAGACACCTTAGGGAAG |
| HSP78 | DQGG008487 | CTTACAGGCGTCTGGGAGTC | CGCCGCATCTTTCTCATTGG |
| 18s | DQGG007047 | TTGAACTTCGTGGAGTCGGG | TTAAACCGTACCCAGGCGAC |

Table S2 Summary of the sequencing data of *Stropharia rugosoannulata* transcriptome at different temperature treatments.

| **Sample** | **Raw reads** | **clean reads** | **Total_Bases** | **Q20%** | **Q30%** | **GC%** | **mapped-reads** | **mapped-rate (%)** |
| --- | --- | --- | --- | --- | --- | --- | --- | --- |
| SR1-1 | 51638984 | 45690174 | 6.83E+09 | 98.46 | 94.68 | 53.25 | 43725496 | 95.7 |
| SR1-2 | 51708012 | 45743758 | 6.84E+09 | 98.54 | 94.91 | 53.45 | 43401677 | 94.88 |
| SR1-3 | 61554248 | 55926608 | 8.36E+09 | 98.5 | 94.81 | 53.13 | 53695136 | 96.01 |
| SR3-1 | 43483918 | 38826916 | 5.8E+09 | 98.58 | 95.12 | 52.91 | 37456325 | 96.47 |
| SR3-2 | 55183406 | 48852968 | 7.3E+09 | 98.57 | 95.08 | 53.28 | 46683896 | 95.56 |
| SR3-3 | 46248746 | 39250736 | 5.86E+09 | 98.47 | 94.79 | 53.31 | 37508003 | 95.56 |
| SR6-1 | 48282310 | 41945944 | 6.27E+09 | 98.61 | 95.2 | 53.53 | 40159046 | 95.74 |
| SR6-2 | 52903614 | 47854960 | 7.15E+09 | 98.49 | 94.71 | 53.08 | 46280531 | 96.71 |
| SR6-3 | 45833258 | 39333154 | 5.88E+09 | 98.4 | 94.48 | 53.42 | 37736227 | 95.94 |
| SR8-1 | 48033812 | 41139102 | 6.14E+09 | 97.64 | 92.66 | 53.29 | 39485310 | 95.98 |
| SR8-2 | 42820102 | 39227696 | 5.86E+09 | 97.46 | 92.26 | 52.68 | 37988100 | 96.84 |
| SR8-3 | 45104014 | 41841364 | 6.24E+09 | 97.69 | 92.81 | 52.81 | 40540096 | 96.89 |

Table S3 GO functional classification of differentially expressed genes.

| **SR1 VS SR6** | |  | | | | |
| --- | --- | --- | --- | --- | --- | --- |
| id | ratio_in_study | ratio_in_pop | p_uncorrected | p_fdr | description | namespace |
| GO:1901615 | 62/1806 | 210/11495 | 1.92E-06 | 0 | organic hydroxy compound metabolic process | biological_process |
| GO:0016791 | 50/1806 | 165/11495 | 2.27E-06 | 0.002 | phosphatase activity | molecular_function |
| GO:0005576 | 66/1806 | 230/11495 | 2.27E-06 | 0.002 | extracellular region | cellular_component |
| GO:0016311 | 54/1806 | 175/11495 | 2.71E-06 | 0.002 | dephosphorylation | biological_process |
| GO:0016491 | 157/1806 | 667/11495 | 3.75E-06 | 0.002 | oxidoreductase activity | molecular_function |
| GO:1901658 | 14/1806 | 25/11495 | 4.25E-06 | 0.002 | glycosyl compound catabolic process | biological_process |
| GO:0055114 | 184/1806 | 822/11495 | 4.48E-06 | 0.002 | oxidation-reduction process | biological_process |
| GO:0007059 | 75/1806 | 287/11495 | 4.97E-06 | 0.004 | chromosome segregation | biological_process |
| GO:0008202 | 30/1806 | 85/11495 | 7.65E-06 | 0.004 | steroid metabolic process | biological_process |
| GO:0005975 | 99/1806 | 406/11495 | 7.65E-06 | 0.004 | carbohydrate metabolic process | biological_process |
| GO:0003824 | 706/1806 | 3837/11495 | 8.19E-06 | 0.006 | catalytic activity | molecular_function |
| GO:0042221 | 260/1806 | 1296/11495 | 1.40E-05 | 0.016 | response to chemical | biological_process |
| GO:0016052 | 55/1806 | 199/11495 | 1.83E-05 | 0.028 | carbohydrate catabolic process | biological_process |
| GO:0009164 | 10/1806 | 16/11495 | 2.87E-05 | 0.044 | nucleoside catabolic process | biological_process |
| GO:0034656 | 10/1806 | 16/11495 | 2.87E-05 | 0.044 | nucleobase-containing small molecule catabolic process | biological_process |
| GO:0050896 | 420/1806 | 2248/11495 | 3.02E-05 | 0.052 | response to stimulus | biological_process |
| GO:0016787 | 325/1806 | 1691/11495 | 3.10E-05 | 0.056 | hydrolase activity | molecular_function |
| GO:0046165 | 35/1806 | 111/11495 | 3.13E-05 | 0.056 | alcohol biosynthetic process | biological_process |
| GO:1990748 | 37/1806 | 120/11495 | 3.68E-05 | 0.07 | cellular detoxification | biological_process |
| GO:0044281 | 234/1806 | 1168/11495 | 3.76E-05 | 0.072 | small molecule metabolic process | biological_process |
| GO:0000272 | 34/1806 | 107/11495 | 4.04E-05 | 0.082 | polysaccharide catabolic process | biological_process |
| GO:0042578 | 53/1806 | 196/11495 | 4.11E-05 | 0.084 | phosphoric ester hydrolase activity | molecular_function |
| GO:0016798 | 50/1806 | 184/11495 | 5.77E-05 | 0.11 | hydrolase activity, acting on glycosyl bonds | molecular_function |
| GO:0016998 | 7/1806 | 9/11495 | 6.28E-05 | 0.12 | cell wall macromolecule catabolic process | biological_process |
| GO:1901617 | 38/1806 | 129/11495 | 7.45E-05 | 0.134 | organic hydroxy compound biosynthetic process | biological_process |
| GO:0044247 | 15/1806 | 34/11495 | 7.83E-05 | 0.142 | cellular polysaccharide catabolic process | biological_process |
| **SR3 VS SR8** | |  | | | | |
| GO:0061077 | 28/2762 | 43/11495 | 3.66E-07 | 0 | chaperone-mediated protein folding | biological_process |
| GO:0034515 | 23/2762 | 36/11495 | 4.24E-07 | 0 | proteasome storage granule | cellular_component |
| GO:0031597 | 27/2762 | 44/11495 | 6.91E-07 | 0 | cytosolic proteasome complex | cellular_component |
| GO:0042255 | 48/2762 | 90/11495 | 1.24E-06 | 0 | ribosome assembly | biological_process |
| GO:0051082 | 56/2762 | 115/11495 | 1.56E-06 | 0 | unfolded protein binding | molecular_function |
| GO:0022626 | 68/2762 | 139/11495 | 1.77E-06 | 0 | cytosolic ribosome | cellular_component |
| GO:0030686 | 68/2762 | 157/11495 | 1.78E-06 | 0 | 90S preribosome | cellular_component |
| GO:0022625 | 40/2762 | 78/11495 | 1.82E-06 | 0 | cytosolic large ribosomal subunit | cellular_component |
| GO:0000463 | 39/2762 | 71/11495 | 1.84E-06 | 0 | maturation of LSU-rRNA from tricistronic rRNA transcript (SSU-rRNA, 5.8S rRNA, LSU-rRNA) | biological_process |
| GO:0006457 | 72/2762 | 171/11495 | 2.03E-06 | 0 | protein folding | biological_process |
| GO:0044445 | 122/2762 | 251/11495 | 2.03E-06 | 0 | cytosolic part | cellular_component |
| GO:0000470 | 43/2762 | 80/11495 | 2.04E-06 | 0 | maturation of LSU-rRNA | biological_process |
| GO:0042273 | 75/2762 | 165/11495 | 2.12E-06 | 0 | ribosomal large subunit biogenesis | biological_process |
| GO:0002181 | 126/2762 | 278/11495 | 2.26E-06 | 0 | cytoplasmic translation | biological_process |
| GO:0003735 | 67/2762 | 154/11495 | 2.41E-06 | 0 | structural constituent of ribosome | molecular_function |
| GO:0022618 | 112/2762 | 288/11495 | 2.41E-06 | 0 | ribonucleoprotein complex assembly | biological_process |
| GO:0030687 | 64/2762 | 137/11495 | 2.48E-06 | 0 | preribosome, large subunit precursor | cellular_component |
| GO:0005840 | 90/2762 | 209/11495 | 2.50E-06 | 0 | ribosome | cellular_component |
| GO:0000466 | 60/2762 | 143/11495 | 2.50E-06 | 0 | maturation of 5.8S rRNA from tricistronic rRNA transcript (SSU-rRNA, 5.8S rRNA, LSU-rRNA) | biological_process |
| GO:0044391 | 75/2762 | 175/11495 | 2.64E-06 | 0 | ribosomal subunit | cellular_component |
| GO:0070925 | 105/2762 | 265/11495 | 2.69E-06 | 0 | organelle assembly | biological_process |
| GO:0000502 | 35/2762 | 69/11495 | 2.78E-06 | 0 | proteasome complex | cellular_component |
| GO:0000460 | 61/2762 | 144/11495 | 2.80E-06 | 0 | maturation of 5.8S rRNA | biological_process |
| GO:0051785 | 52/2762 | 116/11495 | 2.80E-06 | 0 | positive regulation of nuclear division | biological_process |
| GO:0062033 | 44/2762 | 96/11495 | 3.03E-06 | 0 | positive regulation of mitotic sister chromatid segregation | biological_process |
| GO:0006412 | 148/2762 | 380/11495 | 3.11E-06 | 0 | translation | biological_process |
| GO:0030684 | 134/2762 | 304/11495 | 3.16E-06 | 0 | preribosome | cellular_component |
| GO:0022624 | 18/2762 | 27/11495 | 3.20E-06 | 0 | proteasome accessory complex | cellular_component |
| GO:0005838 | 18/2762 | 27/11495 | 3.20E-06 | 0 | proteasome regulatory particle | cellular_component |
| **SR1 VS SR3** | |  | | | | |
| GO:0022857 | 190/1425 | 620/11495 | 3.46E-08 | 4.88E-05 | transmembrane transporter activity | molecular_function |
| GO:0006820 | 142/1425 | 430/11495 | 6.50E-08 | 7.63E-05 | anion transport | biological_process |
| GO:0098656 | 120/1425 | 346/11495 | 8.89E-08 | 8.95E-05 | anion transmembrane transport | biological_process |
| GO:0005215 | 204/1425 | 690/11495 | 9.94E-08 | 8.76E-05 | transporter activity | molecular_function |
| GO:0019748 | 67/1425 | 153/11495 | 1.20E-07 | 9.38E-05 | secondary metabolic process | biological_process |
| GO:0008514 | 103/1425 | 285/11495 | 1.55E-07 | 0.000109 | organic anion transmembrane transporter activity | molecular_function |
| GO:0008509 | 110/1425 | 312/11495 | 1.61E-07 | 0.000103 | anion transmembrane transporter activity | molecular_function |
| GO:0015291 | 96/1425 | 259/11495 | 1.63E-07 | 9.59E-05 | secondary active transmembrane transporter activity | molecular_function |
| GO:0042493 | 161/1425 | 522/11495 | 3.17E-07 | 0.000172 | response to drug | biological_process |
| GO:0042221 | 341/1425 | 1318/11495 | 4.67E-07 | 0.000235 | response to chemical | biological_process |
| GO:0006811 | 189/1425 | 645/11495 | 5.31E-07 | 0.000249 | ion transport | biological_process |
| GO:0022804 | 133/1425 | 415/11495 | 6.92E-07 | 0.000305 | active transmembrane transporter activity | molecular_function |
| GO:0034220 | 161/1425 | 543/11495 | 2.32E-06 | 0.000962 | ion transmembrane transport | biological_process |
| GO:0015301 | 66/1425 | 167/11495 | 2.64E-06 | 0.001033 | anion:anion antiporter activity | molecular_function |
| GO:0140323 | 66/1425 | 167/11495 | 2.64E-06 | 0.000978 | solute:anion antiporter activity | molecular_function |
| GO:1990544 | 64/1425 | 160/11495 | 2.72E-06 | 0.000958 | mitochondrial ATP transmembrane transport | biological_process |
| GO:0005347 | 64/1425 | 160/11495 | 2.72E-06 | 0.000912 | ATP transmembrane transporter activity | molecular_function |
| GO:0140021 | 64/1425 | 160/11495 | 2.72E-06 | 0.000871 | mitochondrial ADP transmembrane transport | biological_process |
| GO:0005471 | 64/1425 | 160/11495 | 2.72E-06 | 0.000833 | ATP:ADP antiporter activity | molecular_function |
| GO:0055085 | 203/1425 | 727/11495 | 3.18E-06 | 0.000934 | transmembrane transport | biological_process |
| GO:0015866 | 64/1425 | 162/11495 | 3.77E-06 | 0.001063 | ADP transport | biological_process |
| GO:0015217 | 64/1425 | 162/11495 | 3.77E-06 | 0.001022 | ADP transmembrane transporter activity | molecular_function |
| GO:0000295 | 65/1425 | 166/11495 | 4.01E-06 | 0.001048 | adenine nucleotide transmembrane transporter activity | molecular_function |
| GO:0005346 | 65/1425 | 166/11495 | 4.01E-06 | 0.00101 | purine ribonucleotide transmembrane transporter activity | molecular_function |
| GO:0015297 | 76/1425 | 207/11495 | 4.30E-06 | 0.001045 | antiporter activity | molecular_function |
| GO:0015216 | 65/1425 | 167/11495 | 4.70E-06 | 0.001105 | purine nucleotide transmembrane transporter activity | molecular_function |
| **SR6 VS SR8** | |  | | | | |
| GO:0000295 | 73/1703 | 166/11495 | 9.71E-06 | 0.03761 | adenine nucleotide transmembrane transporter activity | molecular_function |
| GO:0005346 | 73/1703 | 166/11495 | 9.71E-06 | 0.025074 | purine ribonucleotide transmembrane transporter activity | molecular_function |
| GO:0016491 | 217/1703 | 667/11495 | 1.07E-05 | 0.020744 | oxidoreductase activity | molecular_function |
| GO:0015216 | 73/1703 | 167/11495 | 1.14E-05 | 0.017685 | purine nucleotide transmembrane transporter activity | molecular_function |
| GO:0042910 | 96/1703 | 242/11495 | 1.28E-05 | 0.016555 | xenobiotic transmembrane transporter activity | molecular_function |
| GO:1990544 | 70/1703 | 160/11495 | 1.68E-05 | 0.018624 | mitochondrial ATP transmembrane transport | biological_process |
| GO:0140021 | 70/1703 | 160/11495 | 1.68E-05 | 0.016296 | mitochondrial ADP transmembrane transport | biological_process |
| GO:0005347 | 70/1703 | 160/11495 | 1.68E-05 | 0.014485 | ATP transmembrane transporter activity | molecular_function |
| GO:0005471 | 70/1703 | 160/11495 | 1.68E-05 | 0.013037 | ATP:ADP antiporter activity | molecular_function |
| GO:0008509 | 116/1703 | 312/11495 | 1.79E-05 | 0.012624 | anion transmembrane transporter activity | molecular_function |
| GO:0015893 | 105/1703 | 276/11495 | 2.11E-05 | 0.01361 | drug transport | biological_process |
| GO:0072530 | 73/1703 | 171/11495 | 2.14E-05 | 0.012761 | purine-containing compound transmembrane transport | biological_process |
| GO:0015217 | 70/1703 | 162/11495 | 2.31E-05 | 0.012805 | ADP transmembrane transporter activity | molecular_function |
| GO:0015866 | 70/1703 | 162/11495 | 2.31E-05 | 0.011952 | ADP transport | biological_process |
| GO:0015931 | 142/1703 | 405/11495 | 2.37E-05 | 0.011474 | nucleobase-containing compound transport | biological_process |
| GO:1901679 | 73/1703 | 172/11495 | 2.49E-05 | 0.01137 | nucleotide transmembrane transport | biological_process |
| GO:0015215 | 73/1703 | 172/11495 | 2.49E-05 | 0.010739 | nucleotide transmembrane transporter activity | molecular_function |
| GO:0008514 | 107/1703 | 285/11495 | 2.75E-05 | 0.011199 | organic anion transmembrane transporter activity | molecular_function |
| GO:0015301 | 71/1703 | 167/11495 | 3.07E-05 | 0.011904 | anion:anion antiporter activity | molecular_function |
| GO:0140323 | 71/1703 | 167/11495 | 3.07E-05 | 0.011338 | solute:anion antiporter activity | molecular_function |
| GO:0015297 | 83/1703 | 207/11495 | 3.65E-05 | 0.012842 | antiporter activity | molecular_function |
| GO:0015605 | 75/1703 | 181/11495 | 3.66E-05 | 0.012343 | organophosphate ester transmembrane transporter activity | molecular_function |
| GO:0015291 | 98/1703 | 259/11495 | 4.69E-05 | 0.015135 | secondary active transmembrane transporter activity | molecular_function |
| GO:0098656 | 123/1703 | 346/11495 | 5.18E-05 | 0.016056 | anion transmembrane transport | biological_process |
| GO:1901505 | 76/1703 | 187/11495 | 5.42E-05 | 0.016163 | carbohydrate derivative transmembrane transporter activity | molecular_function |
| GO:0015868 | 75/1703 | 185/11495 | 6.46E-05 | 0.018545 | purine ribonucleotide transport | biological_process |
| GO:0051503 | 75/1703 | 185/11495 | 6.46E-05 | 0.017882 | adenine nucleotide transport | biological_process |
| GO:0015865 | 75/1703 | 186/11495 | 7.42E-05 | 0.01982 | purine nucleotide transport | biological_process |
| GO:0015932 | 75/1703 | 186/11495 | 7.42E-05 | 0.019159 | nucleobase-containing compound transmembrane transporter activity | molecular_function |

Table S4 KEGG pathways enrichment analysis of differentially expressed genes.

| **SR1 VS SR6** | |  |  |  |  |  |  |
| --- | --- | --- | --- | --- | --- | --- | --- |
| layer1 | layer2 | layer3 | ID | Input.number | Background.number | pvalue | FDR |
| 1. Metabolism | 1.0 Global and overview maps | Microbial metabolism in diverse environments | ko01120 | 55 | 190 | 0.00036 | 0.115118 |
| 1. Metabolism | 1.11 Xenobiotics biodegradation and metabolism | Aminobenzoate degradation | ko00627 | 8 | 15 | 0.002732 | 0.437187 |
| 1. Metabolism | 1.3 Lipid metabolism | Fatty acid degradation | ko00071 | 10 | 22 | 0.003712 | 0.395943 |
| 1. Metabolism | 1.1 Carbohydrate metabolism | Pentose and glucuronate interconversions | ko00040 | 9 | 19 | 0.004183 | 0.334663 |
| 5. Organismal Systems | 5.2 Endocrine system | PPAR signaling pathway | ko03320 | 9 | 20 | 0.006358 | 0.406912 |
| 1. Metabolism | 1.0 Global and overview maps | Degradation of aromatic compounds | ko01220 | 11 | 27 | 0.006562 | 0.349992 |
| 2. Genetic Information Processing | 2.4 Replication and repair | DNA replication | ko03030 | 14 | 38 | 0.006606 | 0.301978 |
| 1. Metabolism | 1.3 Lipid metabolism | Steroid biosynthesis | ko00100 | 8 | 17 | 0.00724 | 0.289586 |
| 1. Metabolism | 1.1 Carbohydrate metabolism | Butanoate metabolism | ko00650 | 7 | 14 | 0.007993 | 0.284193 |
| 1. Metabolism | 1.0 Global and overview maps | Metabolic pathways | ko01100 | 187 | 857 | 0.00903 | 0.288968 |
| 1. Metabolism | 1.5 Amino acid metabolism | Glycine, serine and threonine metabolism | ko00260 | 17 | 52 | 0.011336 | 0.329769 |
| 1. Metabolism | 1.8 Metabolism of cofactors and vitamins | Porphyrin and chlorophyll metabolism | ko00860 | 7 | 15 | 0.012596 | 0.33589 |
| 1. Metabolism | 1.11 Xenobiotics biodegradation and metabolism | Metabolism of xenobiotics by cytochrome P450 | ko00980 | 7 | 15 | 0.012596 | 0.310052 |
| 1. Metabolism | 1.1 Carbohydrate metabolism | Pentose phosphate pathway | ko00030 | 9 | 22 | 0.013178 | 0.301221 |
| 1. Metabolism | 1.6 Metabolism of other amino acids | Taurine and hypotaurine metabolism | ko00430 | 4 | 6 | 0.013337 | 0.284529 |
| 1. Metabolism | 1.11 Xenobiotics biodegradation and metabolism | Chloroalkane and chloroalkene degradation | ko00625 | 5 | 9 | 0.014782 | 0.295643 |
| 1. Metabolism | 1.11 Xenobiotics biodegradation and metabolism | Naphthalene degradation | ko00626 | 8 | 19 | 0.015825 | 0.29788 |
| 2. Genetic Information Processing | 2.4 Replication and repair | Mismatch repair | ko03430 | 9 | 24 | 0.024299 | 0.431978 |
| **SR3 VS SR8** | |  | | | | | |
| layer1 | layer2 | layer3 | ID | Input.number | Background.number | pvalue | FDR |
| 2. Genetic Information Processing | 2.3 Folding, sorting and degradation | Proteasome | ko03050 | 27 | 32 | 8.06E-11 | 2.76E-08 |
| 2. Genetic Information Processing | 2.2 Translation | Ribosome | ko03010 | 48 | 91 | 1.04E-06 | 0.000178 |
| 2. Genetic Information Processing | 2.2 Translation | Ribosome biogenesis in eukaryotes | ko03008 | 29 | 58 | 0.000465 | 0.052983 |
| 1. Metabolism | 1.3 Lipid metabolism | Steroid biosynthesis | ko00100 | 11 | 17 | 0.002131 | 0.145763 |
| 1. Metabolism | 1.5 Amino acid metabolism | Arginine and proline metabolism | ko00330 | 15 | 27 | 0.003003 | 0.171147 |
| 1. Metabolism | 1.11 Xenobiotics biodegradation and metabolism | Styrene degradation | ko00643 | 6 | 8 | 0.0088 | 0.429919 |
| 1. Metabolism | 1.11 Xenobiotics biodegradation and metabolism | Aminobenzoate degradation | ko00627 | 9 | 15 | 0.01102 | 0.471119 |
| 4. Cellular Processes | 4.3 Cellular community - eukaryotes | Signaling pathways regulating pluripotency of stem cells | ko04550 | 6 | 9 | 0.020016 | 0.760624 |
| 3. Environmental Information Processing | 3.2 Signal transduction | MAPK signaling pathway - plant | ko04016 | 5 | 7 | 0.023499 | 0.803661 |
| 1. Metabolism | 1.5 Amino acid metabolism | Tryptophan metabolism | ko00380 | 14 | 30 | 0.027153 | 0.773861 |
| 2. Genetic Information Processing | 2.1 Transcription | Spliceosome | ko03040 | 32 | 83 | 0.032272 | 0.849009 |
| 1. Metabolism | 1.8 Metabolism of cofactors and vitamins | Porphyrin and chlorophyll metabolism | ko00860 | 8 | 15 | 0.038434 | 0.938876 |
| 1. Metabolism | 1.1 Carbohydrate metabolism | Pyruvate metabolism | ko00620 | 17 | 40 | 0.042081 | 0.95944 |
| 1. Metabolism | 1.8 Metabolism of cofactors and vitamins | Biotin metabolism | ko00780 | 4 | 6 | 0.06018 | 0.999988 |
| 3. Environmental Information Processing | 3.2 Signal transduction | TNF signaling pathway | ko04668 | 4 | 6 | 0.06018 | 0.999988 |
| 5. Organismal Systems | 5.2 Endocrine system | Renin-angiotensin system | ko04614 | 4 | 6 | 0.06018 | 0.999988 |
| 5. Organismal Systems | 5.1 Immune system | Toll-like receptor signaling pathway | ko04620 | 4 | 6 | 0.06018 | 0.980068 |
| 1. Metabolism | 1.0 Global and overview maps | Biosynthesis of antibiotics | ko01130 | 72 | 214 | 0.060865 | 0.946179 |
| **SR1 VS SR3** | |  | | | | | |
| layer1 | layer2 | layer3 | ID | Input.number | Background.number | pvalue | FDR |
| 1. Metabolism | 1.0 Global and overview maps | Microbial metabolism in diverse environments | ko01120 | 52 | 190 | 6.93E-05 | 0.0201 |
| 1. Metabolism | 1.5 Amino acid metabolism | Glycine, serine and threonine metabolism | ko00260 | 20 | 52 | 0.000102 | 0.014733 |
| 1. Metabolism | 1.0 Global and overview maps | Degradation of aromatic compounds | ko01220 | 13 | 27 | 0.000116 | 0.011256 |
| 1. Metabolism | 1.11 Xenobiotics biodegradation and metabolism | Naphthalene degradation | ko00626 | 10 | 19 | 0.000291 | 0.021107 |
| 1. Metabolism | 1.11 Xenobiotics biodegradation and metabolism | Metabolism of xenobiotics by cytochrome P450 | ko00980 | 8 | 15 | 0.001083 | 0.062815 |
| 1. Metabolism | 1.0 Global and overview maps | Carbon metabolism | ko01200 | 30 | 108 | 0.001757 | 0.084913 |
| 1. Metabolism | 1.11 Xenobiotics biodegradation and metabolism | Polycyclic aromatic hydrocarbon degradation | ko00624 | 8 | 16 | 0.001858 | 0.07697 |
| 1. Metabolism | 1.11 Xenobiotics biodegradation and metabolism | Dioxin degradation | ko00621 | 8 | 16 | 0.001858 | 0.067349 |
| 1. Metabolism | 1.2 Energy metabolism | Methane metabolism | ko00680 | 12 | 31 | 0.002319 | 0.074721 |
| 1. Metabolism | 1.11 Xenobiotics biodegradation and metabolism | Aminobenzoate degradation | ko00627 | 7 | 15 | 0.005843 | 0.169455 |
| 4. Cellular Processes | 4.1 Transport and catabolism | Lysosome | ko04142 | 14 | 43 | 0.006546 | 0.172581 |
| 1. Metabolism | 1.2 Energy metabolism | Sulfur metabolism | ko00920 | 7 | 18 | 0.018467 | 0.446292 |
| 1. Metabolism | 1.1 Carbohydrate metabolism | Pentose phosphate pathway | ko00030 | 8 | 22 | 0.01855 | 0.413803 |
| 1. Metabolism | 1.3 Lipid metabolism | Fatty acid degradation | ko00071 | 8 | 22 | 0.01855 | 0.384246 |
| 1. Metabolism | 1.11 Xenobiotics biodegradation and metabolism | Drug metabolism - cytochrome P450 | ko00982 | 5 | 11 | 0.022498 | 0.434954 |
| 5. Organismal Systems | 5.8 Development and regeneration | Axon regeneration | ko04361 | 6 | 15 | 0.024871 | 0.450785 |
| 1. Metabolism | 1.1 Carbohydrate metabolism | Glycolysis / Gluconeogenesis | ko00010 | 12 | 42 | 0.032395 | 0.552616 |
| 1. Metabolism | 1.2 Energy metabolism | Carbon fixation in photosynthetic organisms | ko00710 | 7 | 20 | 0.033467 | 0.53919 |
| **SR6 VS SR8** | |  | | | | | |
| layer1 | layer2 | layer3 | ID | Input.number | Background.number | pvalue | FDR |
| 1. Metabolism | 1.3 Lipid metabolism | Steroid biosynthesis | ko00100 | 13 | 17 | 5.51E-07 | 0.000176 |
| 1. Metabolism | 1.0 Global and overview maps | Metabolic pathways | ko01100 | 198 | 857 | 0.002166 | 0.346515 |
| 1. Metabolism | 1.11 Xenobiotics biodegradation and metabolism | Metabolism of xenobiotics by cytochrome P450 | ko00980 | 8 | 15 | 0.003366 | 0.359088 |
| 1. Metabolism | 1.3 Lipid metabolism | Biosynthesis of unsaturated fatty acids | ko01040 | 6 | 10 | 0.005312 | 0.424951 |
| 5. Organismal Systems | 5.2 Endocrine system | Thyroid hormone synthesis | ko04918 | 8 | 16 | 0.005603 | 0.358563 |
| 1. Metabolism | 1.5 Amino acid metabolism | Glycine, serine and threonine metabolism | ko00260 | 18 | 52 | 0.006747 | 0.359819 |
| 5. Organismal Systems | 5.2 Endocrine system | PPAR signaling pathway | ko03320 | 9 | 20 | 0.007881 | 0.360275 |
| 1. Metabolism | 1.11 Xenobiotics biodegradation and metabolism | Drug metabolism - cytochrome P450 | ko00982 | 6 | 11 | 0.009788 | 0.391525 |
| 1. Metabolism | 1.6 Metabolism of other amino acids | Glutathione metabolism | ko00480 | 10 | 25 | 0.01361 | 0.483925 |
| 1. Metabolism | 1.6 Metabolism of other amino acids | beta-Alanine metabolism | ko00410 | 9 | 22 | 0.016139 | 0.516437 |
| 1. Metabolism | 1.3 Lipid metabolism | Fatty acid degradation | ko00071 | 9 | 22 | 0.016139 | 0.469488 |
| 1. Metabolism | 1.11 Xenobiotics biodegradation and metabolism | Naphthalene degradation | ko00626 | 8 | 19 | 0.019032 | 0.468482 |
| 1. Metabolism | 1.11 Xenobiotics biodegradation and metabolism | Polycyclic aromatic hydrocarbon degradation | ko00624 | 7 | 16 | 0.022241 | 0.508365 |
| 1. Metabolism | 1.11 Xenobiotics biodegradation and metabolism | Dioxin degradation | ko00621 | 7 | 16 | 0.022241 | 0.474474 |
| 1. Metabolism | 1.0 Global and overview maps | Microbial metabolism in diverse environments | ko01120 | 48 | 190 | 0.02538 | 0.507595 |
| 1. Metabolism | 1.0 Global and overview maps | Biosynthesis of secondary metabolites | ko01110 | 79 | 335 | 0.028325 | 0.533167 |
| 5. Organismal Systems | 5.2 Endocrine system | Aldosterone synthesis and secretion | ko04925 | 5 | 10 | 0.028509 | 0.506818 |
| 1. Metabolism | 1.8 Metabolism of cofactors and vitamins | Ubiquinone and other terpenoid-quinone biosynthesis | ko00130 | 4 | 7 | 0.029606 | 0.49862 |
